# Expression and replication of virus-like circular DNA in human cells

**DOI:** 10.1038/s41598-018-21317-w

**Published:** 2018-02-12

**Authors:** Sebastian Eilebrecht, Agnes Hotz-Wagenblatt, Victor Sarachaga, Amelie Burk, Konstantina Falida, Deblina Chakraborty, Ekaterina Nikitina, Claudia Tessmer, Corinna Whitley, Charlotte Sauerland, Karin Gunst, Imke Grewe, Timo Bund

**Affiliations:** 1 German Cancer Research Center, Division of Episomal-Persistent DNA in Cancer and Chronic Diseases, Heidelberg, Germany; 2 German Cancer Research Center, Core Facility Genomics and Proteomics, Heidelberg, Germany; 3 Biosciences Faculty, University of Heidelberg, Heidelberg, Germany; 4 Department of Oncovirology, Research Medical Center of the Russian Academy of Sciences, Tomsk, Russia; 5 Department of Translational Cell and Molecular Biomedicine, Tomsk State University, Tomsk, Russia; 6 German Cancer Research Center, Core Facility Genomics and Proteomics, Monoclonal Antibody Facility, Heidelberg, Germany

## Abstract

The consumption of bovine milk and meat is considered a risk factor for colon- and breast cancer formation, and milk consumption has also been implicated in an increased risk for developing Multiple Sclerosis (MS). A number of highly related virus-like DNAs have been recently isolated from bovine milk and sera and from a brain sample of a MS patient. As a genetic activity of these *Acinetobacter*-related bovine milk and meat factors (BMMFs) is unknown in eukaryotes, we analyzed their expression and replication potential in human HEK293TT cells. While all analyzed BMMFs show transcriptional activity, the MS brain isolate MSBI1.176, sharing homology with a transmissible spongiform encephalopathy-associated DNA molecule, is transcribed at highest levels. We show expression of a replication-associated protein (Rep), which is highly conserved among all BMMFs, and serological tests indicate a human anti-Rep immune response. While the cow milk isolate CMI1.252 is replication-competent in HEK293TT cells, replication of MSBI1.176 is complemented by CMI1.252, pointing at an interplay during the establishment of persistence in human cells. Transcriptome profiling upon BMMF expression identified host cellular gene expression changes related to cell cycle progression and cell viability control, indicating potential pathways for a pathogenic involvement of BMMFs.

## Introduction

The consumption of red meat has been consistently reported to be a risk factor for developing colorectal cancer^1–4^. Recent studies also linked an increased breast cancer risk to the long-term consumption of red meat and cow milk^5–8^. The breast cancer risk of individuals with lactose intolerance is significantly lower than in lactose-tolerant siblings and parents^9^, pointing at milk or dairy product consumption as risk factors. With only few exceptions, epidemiological data exhibit a remarkable concordance in colon- and breast cancer incidence worldwide and both could be linked to the availability and consumption of meat and/or dairy products of bovine origin^10^,^11^. Also the risk of developing Multiple Sclerosis (MS) has been repeatedly reported to correlate with the consumption of non-pasteurized cow milk^12–14^, the consumption of cream and butter as well as the regional bovine density per inhabitant^14^. Moreover, a variety of studies point at a protective effect of long-term breast feeding against developing MS^15–17^. In the case of red meat, carcinogenic aromatic carbohydrates and nitrosamine derivatives arising during the process of broiling or frying have been suggested to be responsible for the increased cancer risk^18–20^. However, such chemicals are also generated during the processing of poultry and fish^19–22^. Long-time consumption of both has not been linked to an increased risk for these cancers. This suggests such carcinogens at least not being solely responsible for an increased risk for developing cancer. These observations together with the above mentioned epidemiological data have led to a proposed model that species-specific infectious agents of bovine origin - bovine milk and meat factors (BMMFs) – significantly contribute to the etiology of these common human diseases^10,11,23,24^.

A variety of virus-like circular DNAs of BMMFs was recently isolated from commercially available milk (CMIs: Cow Milk Isolates) as well as from serum samples from healthy cattle (HCBIs: Healthy Cattle Blood Isolates)^25–27^, exhibiting a high degree of homology to the transmissible spongiform encephalopathy (TSE)-associated circular DNA isolates Sphinx 1.76 and Sphinx 2.36 at the nucleotide level^28^. Closely related circular DNA molecules were isolated from a brain sample of a MS patient (MSBIs: Multiple Sclerosis Brain Isolates)^25^. All isolates are characterized by a highly conserved A/T-rich region followed by an iteron-like repeat region of 3 × 22 bp + 17/18 bp tandem repeats upstream of the Rep open reading frame (ORF)^25^. Both features, the A/T-rich region as well as the iteron-like repeats are frequently found at the origin of replication of bacterial plasmids, being essential binding sites for the corresponding Rep protein in order to initiate replication^29,30^. Unlike the Rep ORFs, the non-coding regions, which contain the replication-initiating iterons and likely also transcription-regulatory sequences, show less sequence homology to *Acinetobacter baumannii* plasmids, which may point at a potential adaptation for genetic activity in eukaryotes^31^. Additional HCBIs and isolates from serum samples of MS patients (MSSIs: Multiple Sclerosis Serum Isolates) showed similarities to Gemycircular viruses and bacterial plasmids of the species *Psychrobacter*^32,33^. A detailed characterization of all above-mentioned isolates is given by zur Hausen *et al*.^11^.

As a genetic activity of the prokaryote-derived BMMFs and related agents in eukaryotes is unknown to date, we here investigated the expression of selected BMMFs in a human cell system. Moreover, we analyzed whether these agents are replication-competent in human cells. In addition, a helper effect of the cow milk isolate CMI1.252 was noted for the replication of the MS brain isolate MSBI1.176. We used transcriptome profiling in order to investigate BMMF-host cell interactions at the gene expression level, which may give indications for a mode of action during a potential involvement of BMMFs in pathogenesis.

## Results

### Transcriptional activity of BMMFs and related MS brain isolates in HEK293TT cells

HEK293TT cells, expressing the simian virus 40 (SV40) small T antigen and selective for high level expression of SV40 large T antigen^34,35^ have previously been shown to readily transcribe and replicate small single-stranded DNA viruses, such as Torque Teno Viruses (TTVs)^36^.

As the genome size and organization of the identified BMMFs and related isolates are comparable to those of TTVs, we chose this cell line in order to investigate, whether and to which extent the identified BMMFs can be transcribed in the human system. We focused on CMI3.168 and CMI1.252, a small and a large cow milk isolate, the latter one containing two large ORFs - Rep and the uncharacterized ORF-2 - as well as on the MS brain isolates MSBI1.176 and MSBI2.176.

**Figure 1:**
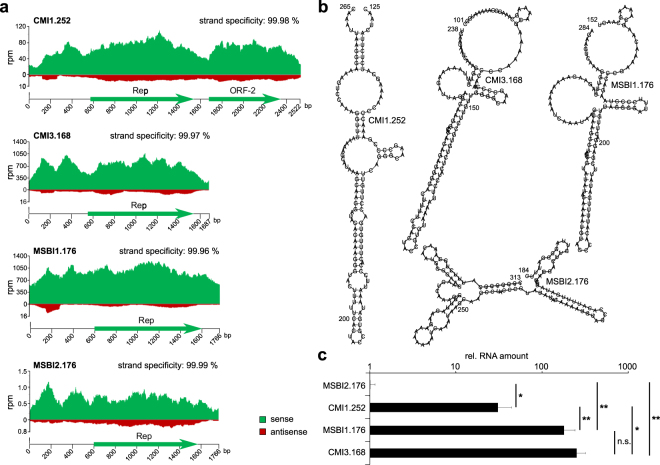
Transcriptional activity of BMMFs and MS brain isolates in HEK293TT cells. (**a**) HEK293TT cells were transfected with circular CMI1.252, CMI3.168, MSBI1.176 and MSBI2.176 genomes. Total RNA was isolated 72 hours post transfection and digested with DNaseI before rRNAs were depleted. The resulting RNA samples were used for strand-specific library generation and next generation sequencing (NGS) of RNA. The strand-specificity of each dataset is given in %. RNA-Seq reads were mapped to each strand of each isolate. Mapped read counts were normalized to the number of total read pairs per run in millions (rpm). Reads mapping to the sense strand are colored in green, reads mapping to the antisense strand are colored in red. ORFs larger than 100 amino acids are indicated. (**b**) Secondary structures of small antisense RNAs generated from the non-coding regions of CMI1.252, CMI3.168, MSBI1.176 and MSBI2.176 as indicated by the RNA-Seq results. Secondary structures have been calculated using RNAfold algorithm^78^. The nucleotide numbers are indicated according to the corresponding full genome sequence. (**c**) RT-qPCR assessment of the transcription levels of CMI1.252, CMI3.168, MSBI1.176 and MSBI2.176 in HEK293TT cells. Circular CMI1.252, CMI3.168, MSBI1.176 or MSBI2.176 was transfected into HEK293TT cells, total RNA was isolated 72 hours post transfection and subjected to reverse transcription using random hexamer primers followed by qPCR quantification of the resulting cDNA using isolate-specific primers. The resulting RNA amounts for each isolate were normalized to the expression of beta actin as a housekeeping gene, before BMMF RNA amounts were normalized to that of MSBI2.176, whose RNA amount was arbitrarily set to 1. The RT-qPCR results shown have been generated from three biological replicates per condition. Statistical significance was calculated using a paired student’s t-test; *p < 0.05, **p < 0.01, n.s. = not significant.

We analyzed transcription of the circularized CMI1.252, CMI3.168, MSBI1.176 and MSBI2.176 genomes 72 hours post transfection in HEK293TT cells by RT-qPCR using specific primers for each genome in order to assess RNA quality (Figure S1A, left). Except for MSBI2.176, all analyzed isolates showed negligible DNA contaminations below 0.2% as identified by the comparion of –RT and + RT qPCR signals. To investigate whether the relative signal of 5% in the –RT reaction of MSBI2.176 originates from a lower RNA quality or whether these findings point at a lower transcriptional activity of MSBI2.176, we further quantified relative differences in beta actin signal between –RT and + RT samples of all isolates (Figure S1A, right). Beta actin signals of the –RT reactions were in all cases ≤ 0.02% of the corresponding signals of the + RT reactions, proving an efficient removal of genomic DNA and a comparable RNA quality for all samples and rather pointing at a lower transcription rate of MSBI2.176. To get deeper insights into BMMF transcription patterns, we next performed RNA-Seq analyses. As the nature of potential arising transcripts – e.g. their polyadenylation capacity and status was unknown and some types of cellular and also viral RNAs, such as certain non-coding (nc)RNAs, are not polyadenylated, we chose a rRNA-depletion strategy rather than a poly(A)-enrichment in order to also cover potential non-polyadenylated transcripts. The rRNA-depleted RNA samples were subjected to strand-specific library generation followed by RNA-Seq. The resulting reads were mapped to each strand of the corresponding genome (Fig. 1a). We first tested whether HEK293TT cells endogenously express BMMF-related sequences by mapping RNA-Seq reads of non-transfected cells to each of the analyzed BMMF genomes, revealing no endogenous expression of BMMF-related sequences in this cell line (data not shown). Notably, MSBI2.176 is transcribed at lowest levels with 1 mapped read per million total read pairs (rpm) on average, while CMI1.252 shows an intermediate transcription level of 65 rpm on average. CMI3.168 and MSBI1.176 exhibit highest transcriptional activity with 700 and 927 rpm on average, respectively. In the case of the highly transcribed genomes CMI1.252, CMI3.168 and MSBI1.176, transcription of the sense strand (Fig. 1a, green) by far exceeds antisense transcription (Fig. 1a, red). Only the weakly transcribed genome MSBI2.176 shows a higher proportion of antisense transcription. However, here the overall antisense transcription rate is lower than in all other isolates, suggesting the generally low transcription levels to be responsible for the higher proportion of antisense transcription. For the small CMI3.168, MSBI1.176 and MSBI2.176 genomes, the transcriptome covers the whole genome in sense direction and read patterns are conserved to a high degree. In the case of CMI1.252, each of the two large ORFs is enriched in sense direction read counts, suggesting that both ORFs may be transcribed separately. In antisense orientation, a short defined stretch starting between 290 and 318 bps upstream of the Rep-ORF is transcribed at higher levels in all four isolates. The prediction of the secondary structure of each of these antisense transcripts resulted in a defined hairpin-structure, which is similar in CMI1.252, CMI3.168 and MSBI1.176, but not in MSBI2.176 (Fig. 1b).

To detect splicing events, the STAR mapping algorithm^37^ was used (Figure S1C), but read counts supporting these splicing events were generally low, suggesting that the splicing frequency is close to the limit of detection and might not have functional consequences in the analyzed cell type. Since RNA-Seq analyses revealed differences in transcriptional activity between the analyzed isolates, we assessed their RNA levels at 72 hours post transfection by RT-qPCR in triplicates using beta actin as a housekeeping gene, supporting the observations made by RNA-Seq (Fig. 1c). Notably, in consistence with the observed lack of endogenous BMMF expression by RNA-Seq, no qPCR signals were detected in control reactions using cDNA from non-transfected HEK293TT cells using the BMMF-specific primers (Figure S1B).

### Identification of MSBI1.176 and CMI1.252 mRNA transcripts and proteins

MSBI1.176 has been directly isolated from brain tissue of a MS patient and is most closely related to the TSE-associated circular DNA molecule Sphinx1.76^28^. MSBI1.176 in our studies reveals the highest transcriptional activity. Thus, we started to further investigate the identity of specific transcripts generated from this genome as a representative of the group of BMMFs containing Rep as the only large ORF. CMI1.252 was included for a more detailed transcript analysis representing BMMFs with additional large ORFs.

**Figure 2:**
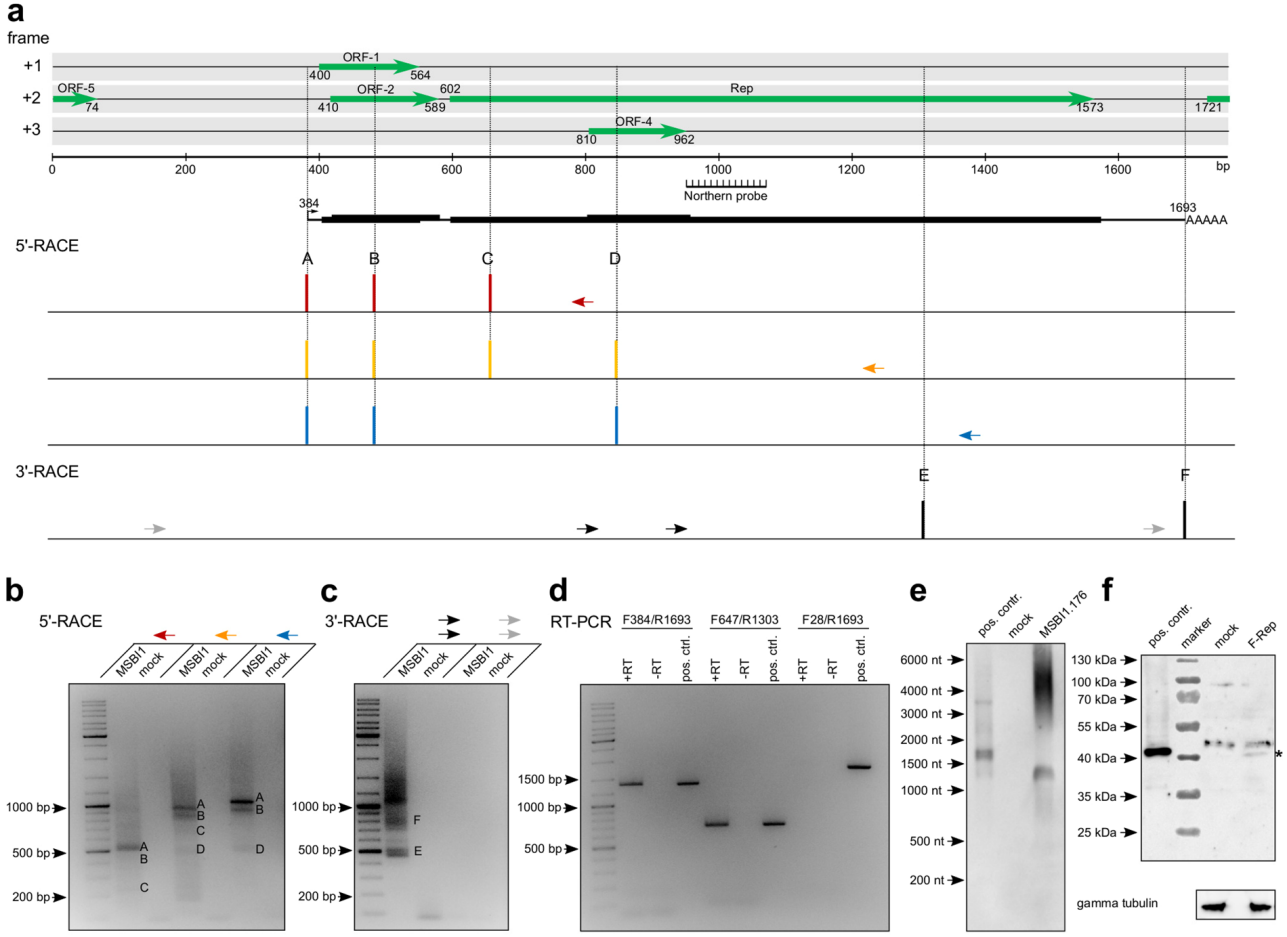
Definition of messenger RNAs transcribed from MSBI1.176. (**a**) Linear representation of the MSBI1.176 genome including ORFs larger than 40 amino acids for the sense strand. The position of the RNA probe for northern blot analyses (Fig. 2e) is indicated. For 5′-RACE, primers located in different regions of the Rep-ORF were used (indicated as red, yellow and blue arrows). Each reproducibly detected transcription start site for each primer is plotted against the location within the genome. For 3′-RACE, nested PCRs were performed with the indicated primers (black and grey arrows). Each reproducibly detected polyadenylation site is plotted against the location within the genome. Putative transcripts arising from the combination of the 5′- and 3′-RACE results, whose existence was supported by northern blot, and their coding potentials are shown. (**b**) Agarose gel image of the MSBI1.176-specific 5′-RACE products obtained by PCR using the primers indicated in panel (a). cDNA of mock-transfected cells was used as a negative control. Specific start sites are indicated by letters according to panel (**a**). (**c**) Agarose gel image of the respective 3′-RACE products obtained by nested PCR using the primers indicated in panel (a). Specific polyadenylation sites are indicated as letters according to panel (a). (**d**) Validation of continuous transcripts using RT-PCR. Total RNA of MSBI1.176-transfected cells was reverse transcribed using oligo-dT primers (+RT) and the resulting cDNA was subjected to PCR reactions using the indicated primer combinations. RT reactions lacking reverse transcriptase served as negative controls (−RT) and PCR reactions using linearized MSBI1.176 DNA as a template served as a positive control.(**e**) Northern blot validation of MSBI1.176 transcripts in HEK293TT cells using an antisense RNA probe as indicated in (**a**). Mock-transfected cells served as a negative control circular MSBI1.176 genome served as a positive control. (**f**) Detection of MSBI1.176 Rep translation in HEK293TT cells transfected with a genome version containing a N-terminal FLAG tag fused to the Rep ORF (F-Rep) using an anti-FLAG antibody. An overexpressed Rep-FLAG fusion protein was used as a positive control and total protein of mock-transfected cells was used as a negative control. Gamma tubulin was used as a loading control.

According to our RNA-Seq results, 99% of MSBI1.176 transcription occurs from the sense strand. Thus, we focused on the sense orientation in order to define mRNA transcripts. 5′-RACE analyses on total RNA of MSBI1.176-transfected HEK293TT cells were performed using primers located in different regions of the Rep ORF in order to identify defined transcription start sites (Fig. 2a,b). All three primer sets consistently detected a frequently used transcription start site at bp 384, located upstream of the iteron repeats and within the A/T-rich region. We identified three additional lower abundance transcription start sites supported by at least two primers at bps 484, 647 and 845 (Fig. 2a,b).

Next, we screened for polyadenylation sites by 3′-RACE using two sets of nested primers, one located in the region covered by the 5′-RACE (black) and a second one located in the region from bp 1655 to 155 (grey) (Fig. 2a,c). The use of the first primer pair indicated that polyadenylation mainly takes place at bp 1303 and 1693, directly downstream of the Rep ORF. We additionally detected larger (>1000 bp) 3′-RACE products using this primer set, which represented fusions of MSBI1.176 (5′-fragment) and human genes (3′-fragment including the polyadenylation site). Such fusion products may be an indication of BMMF integration, but we, however, failed to verify integration events using nested PCRs with specific primers (data not shown) to date. 3′-RACE using the primer pair binding within the region of bp 1655 to 155 did not result in the detection of any polyadenylated transcript (Fig. 2c). Thus, combination of the 5′- and 3′-RACE results suggests the synthesis of polyadenylated transcripts starting at bps 384, 484 and 845 sharing the same polyadenylation site at bp 1693. The longest transcript candidate encodes for ORF-1, ORF-2, Rep and ORF-4 (Fig. 2a).

To analyze the existence of the above-mentioned polyadenylated transcripts, we performed RT-PCR analyses on oligo-dT primed cDNA of MSBI1.176-transfected HEK293TT cells using different primer pairs (Fig. 2d). The sizes of each of the resulting PCR products were identical to positive controls resulting from a PCR amplification of the HindIII (EcoRI)-linearized MSBI1.176 genome, while the -RT control reactions were negative for any PCR product (Fig. 2d). Notably, the PCR reaction of the + RT sample was also negative for additional bands, which could have arisen from frequent splice-products within this region. As this primer combination would detect all concordant splicing events observed in RNA-Seq, we consider these events as low abundance splicing. The use of MSBI1.176 F28, a primer located upstream of the main transcription start site, resulted in no RT-PCR product, while also here the positive control clearly showed a signal, further supporting bp 384 as a major transcription start site for polyadenylated transcripts. We performed northern blot analyses targeting nt 947–1072 by an antisense RNA probe in order to detect MSBI1.176 Rep transcripts (Fig. 2e). While the probe does not hybridize with any cellular RNA (mock), we detected a major transcript of about 1300 nt length in MSBI1.176-transfected cells, which is in line with the 1309 nt transcript constructed from the major TSS and polyadenylation site observed in RACE. To investigate, whether this MSBI1.176-transcribed Rep mRNA is also translated into protein, we performed western blot analyses of HEK293TT cells transfected with a MSBI1.176 genome containing a FLAG-tag fused to the N-terminus of the Rep ORF. Indeed, we observed a faint western blot signal of the same molecular weight as overexpressed MSBI1.176 Rep-FLAG fusion protein, which was used as a positive control (Fig. 2f).

**Figure 3:**
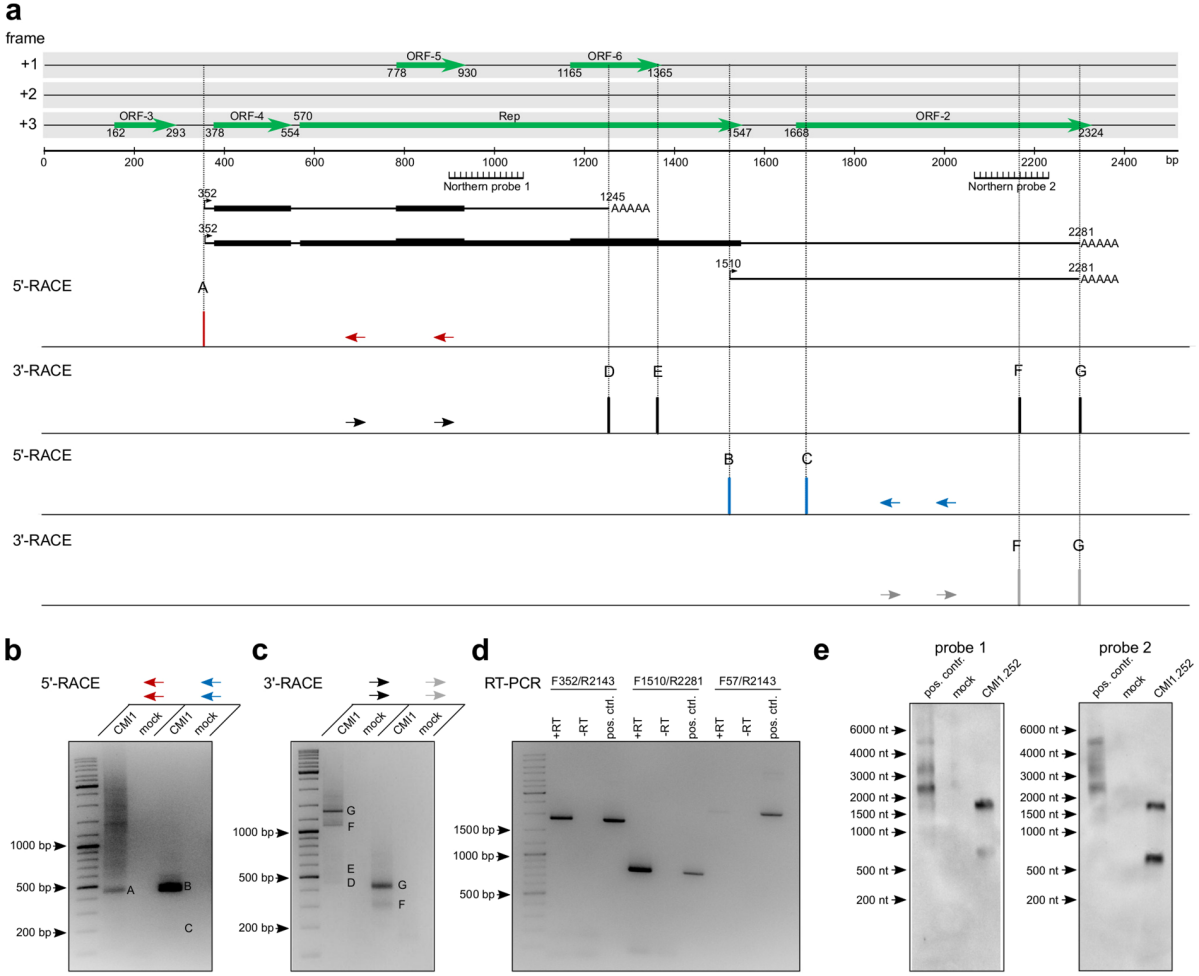
Definition of messenger RNAs transcribed from CMI1.252. (**a**) Linear representation of the CMI1.252 genome including ORFs larger than 40 amino acids for the sense strand. The position of the RNA probes for northern blot analyses (Fig. 3e) are indicated. For 5′-RACE, nested PCR was performed using primers located within the Rep-ORF (indicated as red arrows) and within the ORF-2 (blue arrows). Each reproducibly detected transcription start site for each primer is plotted against the location within the genome. For 3′-RACE, nested PCR was performed using the indicated primers (black and grey arrows). Each reproducibly detected polyadenylation site is plotted against the location within the genome. Transcripts arising from the combination of the 5′- and 3′-RACE results, whose existence was supported by northern blots, as well as their coding potentials are shown. (**b**) Agarose gel image of the CMI1.252-specific 5′-RACE products obtained by PCR using the different primers indicated in panel (a). cDNA of mock-transfected cells was used as a negative control. Specific start sites are indicated by letters according to panel (a). (**c**) Agarose gel image of the respective 3′-RACE products obtained by nested PCR using the primers indicated in panel (a). Specific polyadenylation sites are indicated as letters according to panel (a). (**d**) Validation of continuous transcripts using RT-PCR. Total RNA of CMI1.252-transfected cells was reverse transcribed using oligo-dT primers (+RT) and the resulting cDNA was subjected to PCR reactions using the indicated primer combinations. RT reactions lacking reverse transcriptase was used as negative controls (−RT) and PCR reactions using linearized CMI1.252 DNA as a template served as a positive control. (**e**) Northern blot validation of CMI1.252 transcripts in HEK293TT cells. Antisense RNA probes complementary to nt 883–1051 (probe 1) or nt 2074–2264 (probe 2) of CMI1.252 were used to detect transcripts in CMI1.252-transfected HEK293TT cells. Mock-transfected cells served as a negative control and circular CMI1.252 genome served as a positive control.

As CMI1.252 exhibits significantly lower transcription rates when compared to MSBI1.176, nested PCR reactions were used in order to perform both, 5′- and 3′-RACE. Our initial RNA-Seq data suggested that both large ORFs within CMI1.252 – Rep and ORF-2 – are transcribed separately. Thus, we designed two sets of nested 5′- and 3′-RACE primers, each set targeting one of these ORFs. Nested 5′-RACE using primers targeting the Rep ORF (red), resulted in two defined neighboring transcription start sites at bp 352 and 385, the first one being identical to the major start site in MSBI1.176 (bp 384) (Fig. 3a,b). 3′-RACE with the corresponding complementary primers (black) identified four major polyadenylation sites at bps 1245, 1352, 2143 and 2281 (Fig. 3a,c). Targeting ORF-2 with nested 5′-RACE primers for this region (blue) resulted in two frequent transcription start sites at bps 1510 and 1679, while nested 3′-RACE using the corresponding complementary primers (grey) revealed two major polyadenylation sites at bps 2143 and 2281 (Fig. 3a–c). The combination of these 5′- and 3′-RACE results suggested the expression of transcripts starting at bp 352 using different polyadenylation sites at bps 1245, 1352, 2143 and 2281 as well as two transcripts starting at bp 1510 and 1679 and sharing the same polyadenylation site at bp 2281 (Fig. 3a). The longest of these transcript candidates encodes for ORF-4, Rep, ORF-5 and ORF-6 (Fig. 3a). Notably, two RNA candidates potentially expressed from the ORF-2 region would end upstream of the ORF-2 stop codon, thus lacking a significant complete ORF. These RNAs may thus represent long non-coding RNAs.

As for MSBI1.176, the presence of continuous, polyadenylated transcripts as assembled from the 5′- and 3′-RACE was analyzed by RT-PCR analyses of oligo-dT primed cDNA of CMI1.252-transfected HEK293TT cells with different primer pairs (Fig. 3d). The sizes of the resulting PCR products were identical to the full length sizes of the positive control PCRs using HindIII (AccI)-linearized CMI1.252 DNA as a template. As in the case of MSBI1.176, another control PCR reaction using the primers CMI1.252 F57 combined with R2143 resulted in no significant PCR product when compared to the positive control PCR reaction using circular CMI1.252 DNA as a template. As the first primer is located upstream of the transcription start site at bp 351, these findings suggest a specific transcription start site for polyadenylated transcripts at this position. We performed northern blot analyses using two different antisense RNA probes targeting nt 883–1051 (probe 1) or nt 2074–2264 (probe 2) in order to detect transcripts covering Rep and ORF-2, respectively (Fig. 3e). While probe 2 detects two transcripts, one of about 2000 nt and one of about 800 nt, probe 1 hybridizes with the transcript of about 2000 nt and with an RNA migrating slightly higher than 800 nt, which is in line with three major transcripts of 1929 (probe 1 and 2), 771 (probe 2) and 893 nt (probe 1) as constructed from the combined RACE results. Notably, the northern blot reactions do not differentiate between polyadenylated and non-polyadenylated transcripts. Thus, also the detection of non-polyadenylated transcripts using the identified transcription start sites cannot be ruled out. We failed to detect the transcription start site at bp 351 by 5′-RACE using the primers located within the ORF-2 region, likely due to an impairment of the amplification reaction by the high abundance transcription starting at bp 1510, but the corresponding 3′-RACE analyses using primers complementary to the 5′-RACE primers located within the Rep ORF (black primers) clearly reveal transcripts ending predominantly at bp 2281 (Fig. 3c), supporting a long transcript covering Rep and ORF-2. As for MSBI1.176, we analyzed protein expression in HEK293TT cells also for CMI1.252 using the corresponding genomes containing a FLAG-tag fused to the N-terminus of Rep or ORF-2. In neither case we observed a western blot signal, which indicates that for CMI1.252, which shows only intermediate transcriptional activity, protein expression levels may reside below detection limit (data not shown), in particular for the less frequently used transcription start located at bp 351.

### MSBI1.176 Rep antibody response in human plasma samples

**Figure 4:**
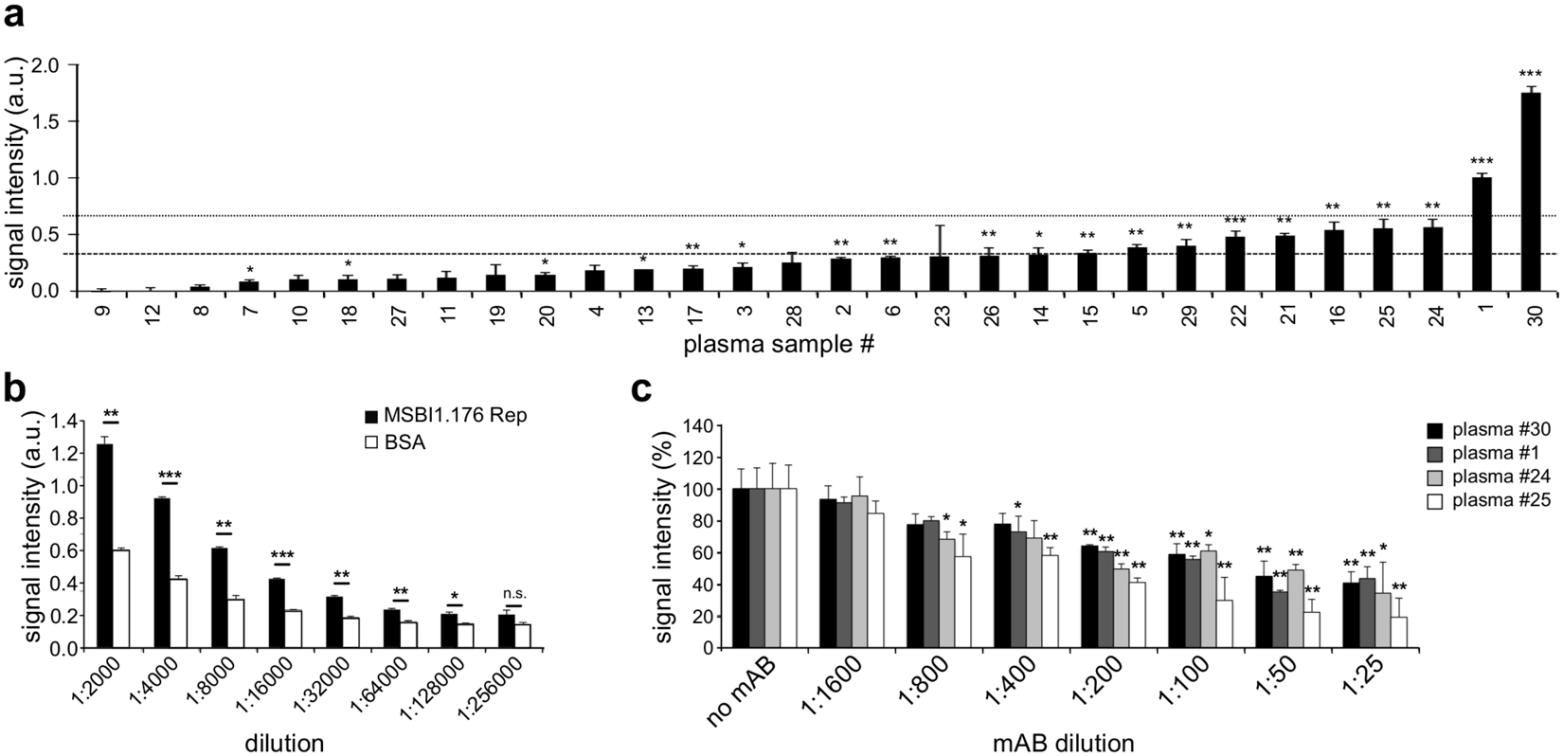
Detection of an antibody response to MSBI1.176 Rep protein in human plasma. (**a**) Thirty human plasma samples were screened for an anti-MSBI1.176 Rep protein antibody response using ELISA assays. The signal intensity is given in arbitrary units (a.u.). Signals are ordered by size and the standard deviation is given based on technical triplicates. The average signal and the sum of average signal and standard deviation of all plasma samples are indicated as dotted lines. Statistical significance as compared to background signal was assessed by student’s t-test from technical triplicates. *p < 0.05; **p < 0.01; ***p < 0.001. (**b**) Quantification of anti-MSBI1.176 Rep protein antibody titers in human plasma. A dilution series (1:1.000 to 1:128.000) of plasma sample # 30 (see panel (a)) was tested for anti-MSBI1.176 Rep reactivity (black) in ELISA assays as compared to reactivity against BSA (white). Statistical significance was assessed by student’s t-test. *p < 0.05; **p < 0.01; ***p < 0.001; n.s. not significant. (**c**) Analysis of anti-Rep specificity of human plasma antibodies. The MSBI1.176 Rep protein coated on ELISA plates was pre-incubated with increasing amounts of an anti-Rep mouse monoclonal antibody pool prior to ELISAs with the human plasma samples #30, #1, #24 and #25. ELISAs with Rep protein lacking a pre-incubation were used as a control reaction (no mAB), whose signal was set to 100%. Statistical significance of the difference to this control reaction were calculated using a paired student’s t-test. *p < 0.05; **p < 0.01.

As we observed MSBI1.176 Rep protein expression in a human cell line, which could serve as an antigen inducing an immune response *in vivo*, we tested a set of 30 plasma samples from healthy human donors for their reactivity against this protein using ELISA assays (Fig. 4a). We observed statistically significant (at least p < 0.05) signal after background subtraction for 20 out of 30 plasma samples, with two samples showing significantly elevated levels of anti-MSBI1.176 Rep antibodies (Fig. 4a). We assessed the antibody titer of the plasma sample showing highest MSBI1.176 Rep reactivity by ELISA assays of serial plasma dilutions (Fig. 4b), showing detectable MSBI1.176 Rep antibodies up to a titer of 1:128.000. In order to prove specificity of the above mentioned serological results, we performed neutralization ELISA experiments using specific anti-Rep mouse monoclonal antibodies. Therefore the coated Rep antigen was incubated with a pool of Rep-specific mouse monoclonal antibodies at different dilutions prior to the application of human plasma samples. Such a pre-incubation should specifically coat the majority of immunogenic Rep epitopes, which should interfere with the specific binding of an antibody from human plasma, while an unspecific binding of antibodies should not be affected. Such a pre-incubation resulted in a signal reduction up to 30% residual signal with increasing amounts of mouse monoclonal anti-Rep antibody pool for the four plasma samples showing the highest anti-Rep response (Fig. 4c), suggesting a specific anti-Rep reactivity.

### CMI1.252 complements MSBI1.176 during DNA replication in HEK293TT cells

Since previous analyses revealed efficient transcription of MSBI1.176, CMI1.252 in HEK293TT cells, we further analyzed whether these agents were also replication-competent in this cell line.

**Figure 5:**
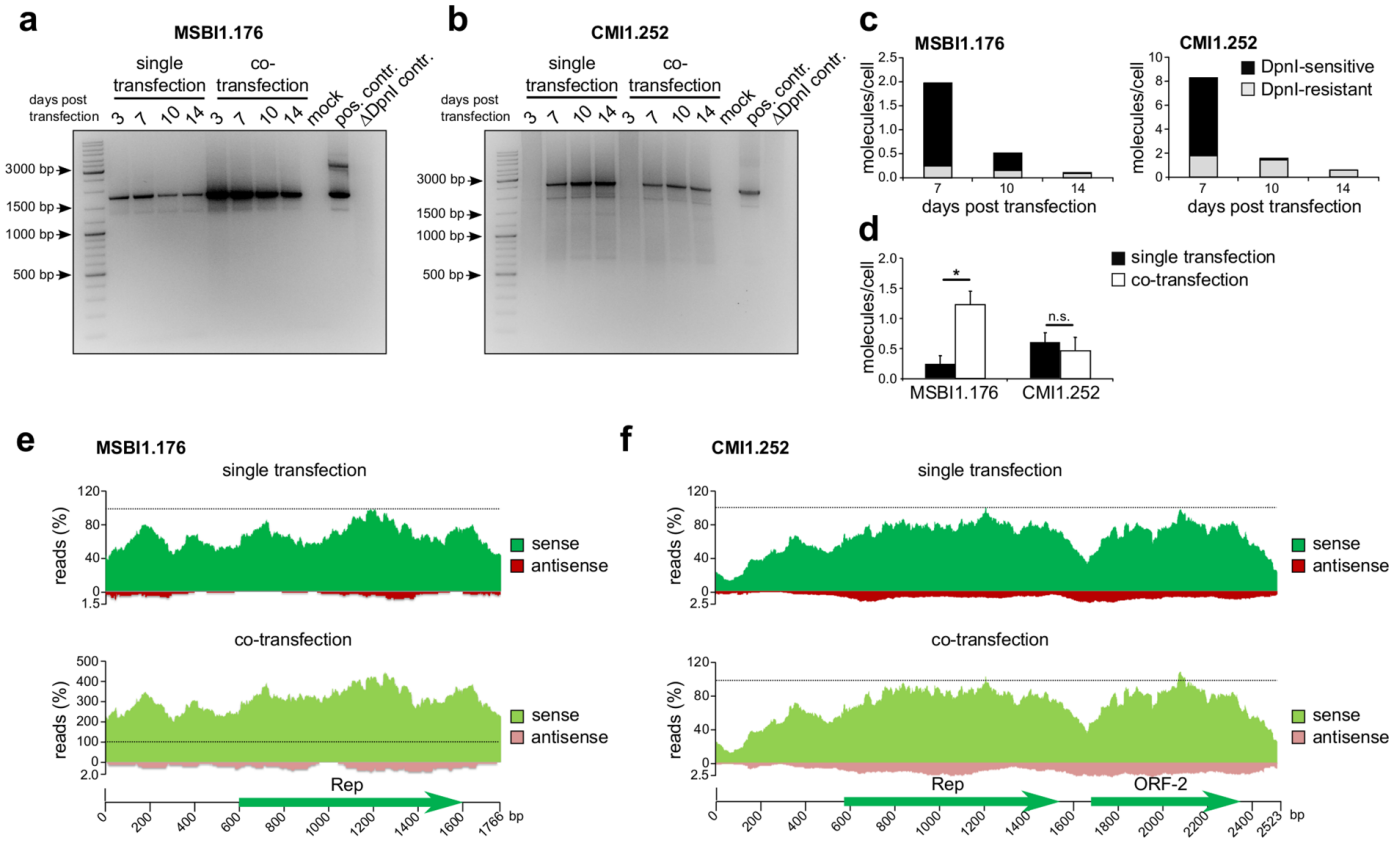
CMI1.252 complements MSBI1.176 during DNA replication in HEK293TT cells. (**a**) HEK293TT cells were co-transfected with equal molecular amounts of circular CMI1.252 and MSBI1.176 (co-transfection) or each isolate alone (single transfection). Total DNA was isolated at days 3, 7, 10 and 14 post transfection and subsequently subjected to extensive DpnI digestion. DpnI-resistant- full length MSBI1.176 DNA was detected for each time point in each condition by long PCR using MSBI1.176-specific back-to-back primers. DNA of mock transfected cells was used as a negative control. 1ng of the circularized *E*.*coli*-generated MSBI1.176 DNA was used as a template for a positive control. DpnI digestion efficiency was tested using the same amount of DpnI-digested circularized *E*.*coli*-generated MSBI1.176 DNA (ΔDpnI control). (**b**) as in (**a**) but detecting DpnI-resistant CMI1.252 DNA by long PCR using CMI1.252-specific back-to-back primers. (**c**) Absolute amounts of DpnI-sensitive (black) and DpnI-resistant (replicated, grey) DNA at days 7, 10 and 14 post transfection of MSBI1.176 and CMI1.252 were quantified by qPCR in biological triplicates. (**d**) Absolute amounts of DpnI-resistant DNA at day 14 post single or co-transfection of MSBI1.176 and CMI1.252 were quantified in biological triplicates by qPCR. Statistical significance was assessed by student’s t-test. *p < 0.05; n.s. not significant. (**e**) and (**f**) Effects of CMI1.252 and MSBI1.176 co-transfection on the transcriptional activity of both isolates. RNA samples harvested at day 3 post single or co-transfection of MSBI1.176 and CMI1.252 were used for strand-specific library generation and next generation sequencing (NGS) of RNA. RNA-Seq reads were mapped to each strand of each isolate and normalized to the maximum read count number in the single conditions, which were arbitrarily set to 100%. Reads mapping to the sense strand are colored in dark green for the single conditions and light green for the co-transfection condition, reads mapping to the antisense strand are colored in dark red for the single conditions and light red for the co-transfection condition. ORFs larger than 100 amino acids are indicated.

Therefore, circularized MSBI1.176 as well as CMI1.252 was transfected into HEK293TT cells. These *E*.*coli*-generated circular MSBI1.176 and CMI1.252 genomes carry an adenine-methylation at the recognition sequence for the DpnI restriction enzyme, allowing for an efficient DpnI digestion. Upon replication in mammalian cells, the DNA loses this methylation, resulting in DpnI resistance, allowing to discriminate input DNA from replicated DNA by DpnI digestion. A DpnI-digestion of the *E*.*coli*-generated input DNAs was used as a template for control reactions, showing complete DpnI digestion of the input DNA in each case (Fig. 5a,b, ΔDpnI control). As the background of genomic DNA present in preparations from transfected cells might influence DpnI digestion efficacy, we compared DpnI digestion efficiency of additional control reactions using input DNA in the absence and the presence of genomic DNA (10 µg of genomic DNA per 10 ng of circularized BMMF genome) by qPCR, proving an efficient digestion of 99% of the input DNA in both conditions (Figure S2A). Non-digested *E*.*coli*-generated input DNAs served as a template for a positive control reaction (Fig. 5a,b, pos. control). Total cellular DNA was harvested at days 3, 7, 10 and 14 post transfection and subsequently subjected to extensive DpnI digestion followed by long PCR reactions using specific back-to-back primers to detect replicated MSBI1.176 and CMI1.252 genomes. At all analyzed time points, DpnI-resistant MSBI1.176 DNA could be detected, with slightly decreasing signals towards later time points, while neither DNA from mock transfected HEK293TT cells nor DpnI-digested input DNA resulted in a long PCR signal, indicating an abortive replication of MSBI1.176 (Fig. 5a). In CMI1.252, the control reactions were also negative and only a very weak signal of DpnI-resistant DNA was observable upon long PCR reaction at day 3. Yet, this signal significantly increased towards later time points, pointing at productive replication of CMI1.252 in HEK293TT cells. We quantified the amounts of DpnI-sensitive (transfected, black) and DpnI-resistant (replicated, grey) DNA at the later time points for each isolate by qPCR (Fig. 5c). We additionally analyzed a replication positive control, consisting of an MSBI1.176 genome containing the SV40 origin of replication (MSBI1.176 SV40) and thus being replication-competent in HEK293TT cells expressing the SV40 large T antigen (Figure S2B). While the amounts of DpnI-sensitive, transfected DNA decreased exponentially over time in all cases, mirroring cell division, the amounts of DpnI-resistant, replicated CMI1.252 and MSBI1.176 DNA decreased by a factor of 3 only when comparing day 7 and day 14 post transfection. At the latest time point, 100% of the detectable CMI1.252 DNA, 80% of the detectable MSBI1.176 DNA and 87% of the detectable MSBI1.176 SV40 was DpnI-resistant. An alternative explaination for an increasing DpnI-resistance of BMMFs may be induced changes in cellular enzymatic activities involved in DNA demethylation, but such changes should simultaneously affect both molecules, MSBI1.176 and CMI1.252 in the same way. As we observe a significantly different course of DpnI-resistance for CMI1.252 when compared to MSBI1.176, we consider the observed increase in DpnI-resistance as indication of DNA replication.

Apart from some variability in the non-coding regions, the main difference between MSBI1.176 and CMI1.252 is the presence of the genomic region containing ORF-2. We analyzed whether trans-acting CMI1.252 gene products from genomic regions different from MSBI1.176, might be responsible for the replication competence of CMI1.252. Amongst others, such a helper effect has been previously reported by Epstein Barr Virus (EBV) for Torque Teno Virus replication^38^. To test this hypothesis, equal molecular amounts of MSBI1.176 and CMI1.252 were co-transfected into HEK293TT cells and subsequently analyzed for replicated MSBI1.176 and CMI1.252 molecules at each time point by long PCR. While the signal of DpnI-resistant CMI1.252 DNA in this co-transfection condition was constant when compared to individual transfections, in co-transfection the DpnI-resistant MSBI1.176 DNA was significantly increased at each of the analyzed time points (Fig. 5a,b). qPCR analyses using DpnI-sensitive, isolate-specific primers to quantify the observed helper effect of CMI1.252 in HEK293TT cells at the latest time point - day 14 post transfection - were performed (Fig. 5d). While numbers of CMI1.252 genomes of about one molecule per two cells were not significantly changed when comparing single and co-transfection, the MSBI1.176 genome copy number in the co-transfection condition was statistically significantly increased from about one molecule per four cells to more than one molecule per cell (Fig. 5d).

We determined whether this effect was also detectable at RNA level and whether it might also involve changes in RNA expression patterns of the individual isolates. Therefore, strand-specific RNA-Seq was performed at day 3 post co-transfection of MSBI1.176 and CMI1.252. These data were compared to transfections of each isolate alone (Fig. 5e,f). In the case of CMI1.252, neither the amount nor the pattern of transcription significantly changed upon co-transfection with MSBI1.176 (Fig. 5f). In contrast, in co-transfection with CMI1.252 an about four-fold increase of transcription rate was noted for MSBI1.176, while the overall transcription pattern remained almost identical (Fig. 5e).

### MSBI1.176 and CMI1.252 significantly co-affect the expression of human target genes

**Figure 6:**
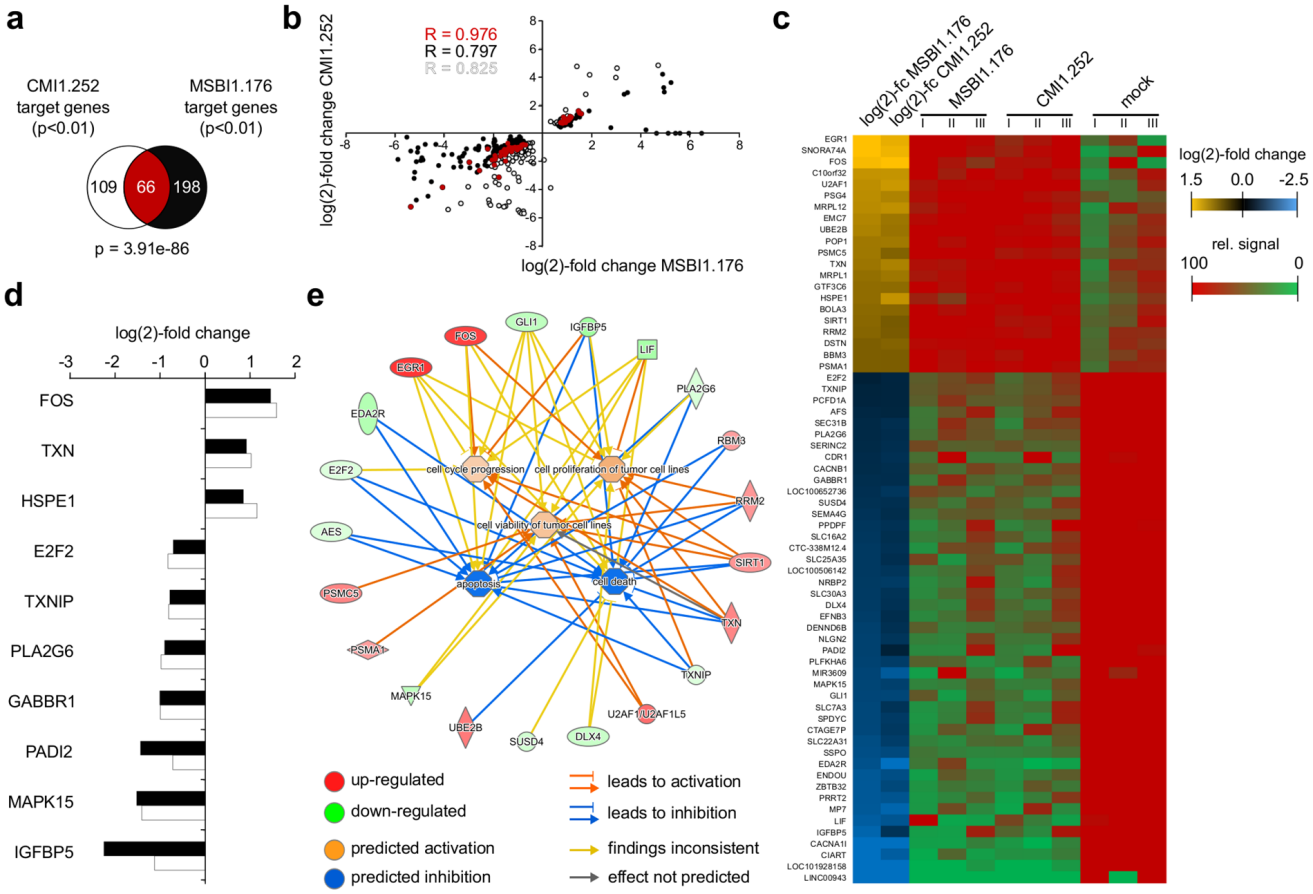
Human gene expression changes upon MSBI1.176 and CMI1.252 expression in HEK293TT cells. (**a**)Venn diagram of the gene sets statistically significantly (p < 0.01) regulated upon MSBI1.176 expression (black) as well as upon CMI1.252 expression (white). The common subset is highlighted in red and the corresponding hypergeometric distribution p-value is given below. Gene numbers for each set are indicated. (**b**) Scatter plot of the genes shown in panel (a) using the same colors. The log(2)-fold change in expression upon MSBI1.176 expression is plotted on the x-axis and the log(2)-fold expression change upon CMI1.252 expression is plotted on the y-axis. The Pearson correlation coefficients (R) for the common subset as well as for the remaining genes of each single target gene set are indicated. (**c**) Heatmap of the common subset of MSBI1.176- and CMI1.252-regulated human genes. The log(2)-fold change in gene expression in both conditions (MSBI1.176 and CMI1.252 expression) is indicated as a color code for the common subset of 66 genes. Up-regulated genes are colored in yellow, down-regulated genes are colored in blue. For each gene, the relative, normalized signal for three biological replicates is indicated as a color code. (**d**) Representative genes co-regulated upon MSBI1.176 and CMI1.252 expression. Expression changes upon MSBI1.176 expression are colored in black, those upon CMI1.252 expression are colored in white. (**e**) Representation of genes involved in regulation of cell cycle progression, proliferation and viability of tumor cell lines, cell death and apoptosis as identified by canonical pathway enrichment analyses of the common subset shown in panels (a) and (c). Genes, whose expression is up-regulated upon BMMF expression are colored in red, those whose expression is down-regulated are colored in green.

As the results supported a specific expression of the BMMFs MSBI1.176 and CMI1.252 in human HEK293TT cells, we investigated effects of these agents on host cell gene expression. Therefore, RNA-Seq data of MSBI1.176- and CMI1.252-transfected HEK293TT cells and corresponding non-transfected control cells were used to identify statistically significantly (p < 0.01) modulated human target gene expression for each isolate. The expression of MSBI1.176 resulted in a total of 264 differentially regulated genes, of which 71 genes were up- and 193 genes were down-regulated (Table S1). In the case of CMI1.252, a total of 175 genes were differentially regulated, out of which 43 were up- and 132 were down-regulated (Table S2). Interestingly, the common subset of human genes differentially expressed upon both MSBI1.176 and CMI1.252 expression comprised a highly significant (Fig. 6a, hypergeometric distribution p-value of 3.91e-86) set of 66 genes – 21 being up- and 45 being down-regulated (Fig. 6b,c), which might represent general targets of BMMFs. A control transfection of pUC19 vector resulted in comparable changes only for two genes of the common subset (PSG4 and CDR1), while the expression of the remaining genes was not significantly changed, supporting that the transfection of DNA in general was not responsible for the observed gene expression changes (data not shown). Furthermore, the vast majority of remaining target genes for each separate condition appeared to be consistently regulated upon expression of the corresponding other isolate, as apparent from a Scatter plot representation of all three gene sets (Fig. 6b). The Pearson correlation coefficient for the common subset, but also for the remaining genes of each single target gene set revealed the expression changes of the human target genes of MSBI1.176 and CMI1.252 to be highly positively correlated (Fig. 6b,d). Canonical pathway enrichment analyses of the common gene subset identified a significant proportion of 21 genes being involved in the regulation of cell cycle progression, proliferation and viability of tumor cell lines, cell death and apoptosis (Fig. 6e).

## Discussion

A number of studies have supported the consumption of milk and dairy products to represent risk factors for the development of certain human diseases such as colon-, breast- and prostate cancer, MS or Parkinson’s disease^10–14,39–45^. A model has been proposed, in which infections with milk- or dairy product-derived agents, abbreviated as BMMFs, contribute to disease onset and/or progression^10,11,23,24^. The recent identification of virus-like small circular DNA molecules provided BMMFs isolated from sera and commercially available milk and dairy products^11,25^. It was striking that molecules of the BMMF group could also be isolated from a lesion of a brain biopsy of a MS patient^25^, providing a possible link between these infections and a neurodegenerative disease. Similar circular DNA molecules have been previously isolated from scrapie-infected murine neuroblastoma cells as well as from scrapie-infected hamster brains and mouse brains infected with Fujisaki-Creutzfeldt-Jakob disease (FU-CJD) strain^28^, further supporting a role in neurodegeneration. However, these findings raised a number of questions, which need to be addressed in order to understand a potential involvement of BMMFs in the onset or the progression of a particular human disease:

1. Can the isolated BMMFs be specifically transcribed/expressed in human cells?
2. Are the expressed BMMF gene products able to evoke a human immune response?
3. Are the BMMFs capable to replicate/persist in human cells?
4. Does BMMF expression alter the gene expression of the host cell? If yes, which genes/pathways are affected?

All BMMFs, including the MSBIs are to a high extent related to sequences of *Acinetobacter baumannii* plasmids, the closest being the plasmid pAB120 (Figure S2A and B)^31^, supporting a bacterial origin. As the MSBIs have been directly isolated from human tissue, it was of high importance to analyze their genetic activity in eukaryotic, and more precisely in human cells. A recent study suggests the expression of the Sphinx 1.76 Rep protein in murine GT1 cells, in mouse and hamster brain samples as well as in human glioblastoma samples using antisera reactive against one peptide of the protein in western blot and immune-histochemical analyses^46^. Though these results suggest that such prokaryote-derived DNA sequences may be transcribed and translated in eukaryotes, a cross-reaction of the generated antisera with an endogenous protein in these studies would still have to be experimentally excluded.

Here, we first assessed the transcriptional activity of BMMFs and related MSBIs in the HEK293TT line^47^. To date only a limited number of studies has analyzed transcription of DNA viruses of comparable genome size, such as adeno-associated virus 2 (AAV2, 4697 bp) or hepatitis B virus (HBV, 3221 bp), using RNA-Seq technology^48,49^. All four analyzed BMMFs were transcribed in HEK293TT cells, though at significantly different levels. To our surprise, the transcriptome of the isloates covered the whole genome of each corresponding BMMF with only very faint read-enrichments within ORFs. However, also the read patterns previously observed for AAV2 or HBV do not show significant enrichments for ORFs^48,49^. Notably, all BMMFs show specific sense transcriptional activity, strongly arguing against spontaneous, undirected transcription. Interestingly, the transcriptional activity of the analyzed isolates in human cells is inversely correlated with their degree of relationship with the bacterial pAB120 plasmid (Figure S3A). The regulatory region (A/T-rich region and iteron-like repeats) of the isolate MSBI2.176 is closest related to that of the *Acinetobacter baumannii* plasmid pAB120 (Figure S3B), suggesting that differences in such regulatory DNA regions may account for significant differences in transcription levels in human cells.

Besides several low frequency transcription start sites (TSS), the most frequently used TSS of MSBI1.176 and CMI1.252 was located 218 bp upstream of the Rep ORF, within the A/T-rich region of the genomes, which is important for plasmid replication^29,50^. In both cases, the region directly upstream of this transcription start site contains several binding sites for transcription factors such as AP-1, HNF-4α or C/EBPα, as identified by Alibaba2 algorithm^51^ (Figure S4). Notably, the sequence of the nascent transcript as well as the putative TATA-box are conserved among different BMMFs and show similarities to nascent transcripts and regulatory sequences of mammalian ribosomal proteins^52^ (Figure S4). Moreover, our RNA-Seq analyses revealed short antisense transcripts of 129 to 140 nt in length arising from a genomic region located 70–100 bp upstream of this transcription start site (see Fig. 1a,b). Such an antisense transcription has been recently observed from regions upstream of active promoters, resulting in transcription start site (TSS)-associated antisense RNAs (TSSa-RNAs)^53–57^, further supporting this locus within the BMMF genomes as active transcription regulatory region in humans. The resulting antisense RNA transcripts – at least in the case of the BMMFs with higher transcriptional activity – can fold into stable stem-loop structures, which show in particular in their apical region similarities among the different isolates (see Fig. 1b). While the function of TSSa-RNAs remains still elusive, the stable stem-loop structures of the particular BMMF antisense transcripts observed here may suggest that they are substrates for Dicer-mediated cleavage and further downstream processing to micro RNAs (miRNAs). However, a detailed functional characterization of these transcripts will need to be addressed in subsequent studies.

In eukaryotes, the maturation process of pre-mRNA to mRNA involves polyadenylation at the 3′-end of the transcript, important for nuclear export, efficient translation as well as for mRNA stability. For MSBI1.176 we identified two major polyadenylation sites located 270 bp upstream and 120 bp downstream of the Rep ORF by RACE analyses. In both cases, the non-canonical polyA signal AAAACA was located 17 and 18 bp upstream of the polyadenylation site, respectively^58^. This non-canonical polyA signal may allow for partial run-through transcription without termination at the corresponding transcription termination site. The resulting non-polyadenylated transcripts may account for RNA-Seq signals in genomic regions located downstream of the polyA signal and would not be detectable in 3′-RACE reactions. In concordance, we observed high molecular weight transcripts in addition to the major polyadenylated transcript using northern blot analyses targeting the Rep ORF. Moreover, we observed MSBI1.176 Rep protein expression, further arguing for proper mRNA polyadenylation.

We used ELISAs to analyze whether BMMF Rep proteins may be capable of inducing a human immune response *in vivo*. We detected an elevated anti-MSBI1.176 Rep immune response in two out of 30 plasma samples from healthy human donors, supporting an immunogenic role of BMMFs and suggesting that an infection with BMMFs or highly related agents may have taken place at some point earlier in life.

An important prerequisite for a potential pathogenic role of BMMFs in human is their ability to replicate or persist in human cells. While replication of MSBI1.176 was abortive in highly proliferating HEK293TT cells, CMI1.252 replication resulted in almost stable genome amounts until day 14 post transfection. As the Rep ORFs of both isolates are virtually identical, we assumed that a CMI1.252 gene product from a different region may account for these observations. Further assuming that this gene product could act in *trans*, a co-expression of both isolates should positively impact MSBI1.176 replication. Indeed, we observed a helper effect of CMI1.252 expression for MSBI1.176 DNA replication, a phenomenon, which has been described for a number of different viruses^39,59,60^ and allows to consider small BMMF molecules as replication-incompetent satellites of the large ones, reminiscent of geminivirus replication^61^.

The pathogenicity of many viruses is mediated by pathogen-host interactions resulting in significant changes in host cellular gene expression and subsequently in phenotypic changes of the cell. Here, we identified a set of significantly regulated BMMF target genes of MSBI1.176 and CMI1.252 (see Fig. 6). Among the up-regulated genes, we observe the proto-oncogene FOS^62^ as well as a set of other cancer-related genes such as HSPE1, TXN and TXNIP. The heat shock protein HSPE1 has been previously suggested to be involved in cancer etiology and is overexpressed in exocervical carcinoma as well as in colon cancer^63–65^. Thioredoxin (TXN) is overexpressed in colorectal carcinoma and its expression rates have been linked to the aggressivity of the tumor^66,67^. Among the target genes, which are repressed upon BMMF expression are the tumor suppressor genes E2F2 and TXNIP as well as the phospholipase PLA2G6, which is frequently mutated in neurodegenerative diseases. E2F2 acts as a tumor suppressor by modulating apoptosis via the suppression of Myc-induced proliferation and tumorigenesis^68,69^, while TXNIP has been shown to act as a tumor suppressor in thyroid cancer^70^. PLA2G6 is a phospholipase, which is involved in hydrolysis of glycerophospholipids. Notably, this gene is frequently mutated in neurodegenerative disorders with high brain iron, such as Parkinson’s disease and Alzheimer’s disease^71,72^. A subset of the genes commonly regulated by MSBI1.176 and CMI1.252 are involved in the regulation of cell cycle progression and have been implicated to be involved in the proliferation and viability of tumor cell lines as well as in cell death and apoptosis (see Fig. 6e), a finding which may link BMMF expression to cellular pathways impaired during cancerogenesis.

Our results prove genetic activity of BMMFs in the eukaryotic, human cell system, observing transcription and translation of the highly conserved Rep gene. Replication competence of CMI1.252 and its helper effect for MSBI1.176 replication could establish a long-term persistence of BMMFs in human cells. BMMF expression results in significant changes in the transcription of host cellular genes involved in fundamental cellular processes, which may provide a mechanism by which BMMFs could contribute to the onset or progression of diseases such as colon-, breast- and prostate cancer or neurodegenerative diseases.

## Methods

### Plasmids and DNA circularization

All healthy cattle blood isolates, MS brain isolates and cow milk isolates have been described previously^11,25,26^. For self-circularization of BMMF genomes, 50 µg of the pUC19-cloned isolates were released from the vector backbone by restriction digestion. The linear BMMF DNA was separated by agarose gel electrophoresis and eluted using the Nucleospin Gel Cleanup kit (Macherey & Nagel). The DNA was ligated in 1xT4 DNA ligase buffer (Fermentas) in a total volume of 9 ml at 4 °C for 48 hours using 30 U T4 DNA ligase (Fermentas). After addition of 4.5 ml of 7.5 M ammonium acetate and 35 ml of 95% ethanol the circular DNA was precipitated at 4 °C over night. Precipitated DNA was washed with 70% ethanol and the self-circularization was tested by restriction digestion. To construct MSBI1.176 FLAG-Rep, CMI1.252 FLAG-Rep and CMI1.252 FLAG-ORF-2 we introduced a single FLAG-tag sequence (DYKDDDDK) directly downstream of the start codon of the Rep ORF/ORF-2 within the pUC19-cloned BMMF genome by mutagenesis PCR.

### Cell culture

HEK293TT cells^35,36^ were maintained in Dulbeccos modified Eagles medium (DMEM) containing 10% fetal calf serum, 1% Glutamax, 1% non-essential amino acids (Invitrogen) and 400 µg/ml hygromycin B (Roche Diagnostics) at 37 °C and 5% CO_2_. For DNA and RNA isolation cells were kept in T25 flasks and transfected at 70–80% confluency with 5 µg of circular DNA using Lipofectamine 2000 (Invitrogen) according to the manufacturer’s instructions. To remove residual DNA, the medium was exchanged by fresh medium 24 hours post transfection. For co-transfections the total DNA amount was split into equimolar amounts of each molecule. For control, mock transfections were performed, in which the cells were treated exactly as mentioned above, but no DNA was added to the transfection mixture.

### DNA isolation

For total DNA isolation of transfected cells, cells were lysed in 0.5% SDS-Tris-EDTA buffer, before RNA was removed by incubation with 10 µg RNase A at 37 °C for 30 minutes and proteins were digested by incubation with 200 µg proteinase K for 1 hour at 50 °C. Subsequently, the DNA was purified by phenol-chloroform extraction and precipitated with ethanol. To remove input DNA for replication studies 10 µg of total DNA were subjected to DpnI digestion in a total volume of 400 µl for 16 hours at 37 °C using 100 U of DpnI (Fermentas). The DpnI-resistant-DNA was subsequently precipitated with ethanol. 200 ng of DpnI-resistant- DNA was used for either qPCR or long PCR reactions.

### RNA isolation

Total RNA of transfected cells was isolated using the miRNeasy kit (Qiagen) according to the manufacturer’s instructions. To remove residual DNA molecules for downstream applications, 100 µg RNA were incubated with 15 U of DNase1 (Promega) in the presence of 150 U RNasin (Promega) at 37 °C for 30 minutes. Subsequently, the RNA was precipitated with ethanol. For the generation of random-primed libraries for RNA-Seq analyses, DNase1-treated RNA was depleted for rRNAs using the RiboZero kit (Illumina) according to the manufacturer’s instructions. During all RNA processing steps the RNA quantity was monitored by Nanodrop technology (Thermo Fisher Scientific) and RNA quality as well as rRNA-depletion efficacy was analyzed by Bioanalyzer technology (Applied Biosystems) according to the manufacturer’s instructions. RNA integrity numbers (RIN) of all processed samples were 8.0 or higher.

### Protein isolation

For total protein isolation, the cells were washed once with PBS followed by direct lysis in SDS-PAGE Lämmli buffer including 0.5 µl/well Benzonase for digestion of DNA.

### Reverse transcription (RT) PCR and long PCR

For cDNA synthesis, 1 µg of DNase1-treated total RNA was reverse transcribed using random hexamer or oligo-dT primers using SMARTScribe Reverse Transcriptase (Clontech) as recommended by the manufacturer. A reaction lacking reverse transcriptase served as a negative control (-RT). Primers used for the detection of cDNAs of polyadenylated BMMF transcripts as well as a detailed procedure are listed in the supplemental methods section.

Long PCR for detection of full length MSBI1.176 or CMI1.252 DNA was performed using DpnI-digested DNA samples and La Taq polymerase (Takara) as recommended by the manufacturer. Back-to-back primers for the detection of BMMFs and a detailed protocol can be found in the supplemental methods section.

### Quantitative PCR (qPCR)

Quantitative PCR (qPCR) analyses were performed using the SYBR Green PCR Master Mix (Applied Biosystems) and a Stratagene Mx3000P qPCR machine (Stratagene) as recommended by the manufacturer. Primers used for the quantification of RT-generated cDNA or DpnI-resistant-, replicated DNA are listed in the supplemental methods section.

### Next generation sequencing of RNA (RNA-Seq)

DNase1-treated, rRNA-depleted total RNA of transfected cells was used for the generation of random-primed, strand-specific cDNA libraries using the TruSeq Stranded Total RNA Library Prep Kit (Illumina) as recommended by the manufacturer. Libraries were sequenced in 125 bp paired-end mode on an Illumina HiSeq2500 sequencer (Illumina) resulting in at least 30 Mio read pairs per sample. Strand specificity of the data was determined by mapping the paired-end data to the mRNA sequences of four highly expressed cellular genes with no known antisense transcription (ACTB, GAPDH, RPL8 and EEF2) using TopHat2 mapping algorithm^73^. RNA-Seq reads of biological triplicates were mapped to two permutated BMMF sequences of each isolate using TopHat2 mapping algorithm and mapped reads were subsequently combined to account for the circular nature of the BMMF genomes. Mapped reads were split into sense and antisense reads using samtools package^74^ and normalized to the total number of read pairs in millions (rpm) in each experiment. STAR mapping algorithm^38^ was used for the detection of putative splicing events. Only splicing events being consistently observed in at least two biological replicates were considered as real events.

For analyzing human gene expression changes, RNA-Seq reads were mapped to the human genome using TopHat2 mapping algorithm and mapped reads were subsequently counted using HtSeq-Count^75^. Differential gene expression was further analyzed by comparing mapped RNA-Seq reads in each biological condition and statistical significance was calculated using the edgeR package applying default settings^76,77^.

Canonical pathway enrichment analyses were performed for statistically significantly regulated gene subsets using Ingenuity Pathway Analysis software as recommended by the manufacturer.

### Rapid amplification of cDNA ends (RACE)

5′- and 3′-RACE analyses were performed using the SMARTer RACE 5′3′ kit (Clontech) as recommended by the manufacturer. A detailed protocol and all primer sequences can be found in the supplemental methods section.

Resulting RACE fragments were separated by agarose gel electrophoresis, eluted using the NucleoSpin gel cleanup kit (Machery & Nagel), subcloned into the pRACE vector (Clontech) as recommended by the manufacturer and identified by conventional Sanger sequencing. For MSBI1.176 a total of 497 clones were sequenced (primer R813: 128 clones, primer R1258: 108 clones, primer R1383: 167 clones, primer F944: 94 clones). For CMI1.252 a total of 384 clones were sequenced (primer R724: 96 clones, primer R1883: 108 clones, primer F894: 120 clones, primer F1976: 60 clones). RACE reactions using cDNAs generated from DNase1-treated total RNA of mock transfected HEK293TT cells were used as negative controls. To exclude PCR artifacts, an additional control was included, in which linearized BMMF DNA was used as a template for RACE reactions.

### Northern blot analyses

For northern blot analyses 5 µg of total cellular RNA were separated on a 1% denaturing Formaldehyde agarose gel and subsequently diffusion-transferred to a Hybond N + Nylon membrane (Amersham). After UV crosslinking, BMMF-derived RNAs were hybridized with specific DIG-labeled RNA probes and upon incubation with anti-Digoxigenin-AP Fab fragments (Roche) signals were detected on a BioRad WesternBlot detection system. A detailed protocol can be found in the supplemental materials section.

### Western blot analyses

Protein samples were separated by SDS-PAGE on Sigma True-PAGE 4–20% precast gels, electro-transferred to nitrocellulose membrane and detected by western blot procedure using M2 anti-FLAG antibody (Sigma) and a corresponding HRP-coupled goat anti-mouse secondary antibody (Dianova) as recommended by the manufacturer. Signals were visualized by incubation with Pierce Femto HRP substrate on a BioRad WesternBlot detection system.

### ELISAs and serology

30 plasma samples from anonymous healthy human donors were analyzed for anti-MSBI1.176 Rep antibodies using ELISAs. ELISA plates (Maxisorp, Thermo Fisher Scientific) were coated with 200 ng/well of purified, denatured MSBI1.176 Rep protein at 4 °C over night. Blocking was performed in superblock assay buffer (Thermo Fisher Scientific) for 2 hours at room temperature. Plasma incubation (serial dilutions starting from a 1:1000 dilution in superblock buffer) was performed for 1 hour at 37 °C in superblock buffer followed by three washing steps with PBS containing 0.1% Tween at room temperature. MSBI1.176 Rep-bound human IgG antibodies were quantified using a HRP-coupled goat anti-human secondary antibody (1:5000 dilution, 1 hour at 37 °C, followed by additional three washing steps with PBS containing 0.1% Tween 20) in triplicates (TMB ELISA substrate, Thermo Fisher Scientific; ELISA stop solution containing 8 M acetic acid and 1 M sulfuric acid, readout at 450 nm). The production of mouse monoclonal anti-Rep antibodies, the purification of MSBI1.176 Rep protein for ELISAs as well as a detailed protocol for neutralization ELISAs can be found in the supplemental materials section.

Informed consent was obtained from all subjects. All experiments were performed in accordance with relevant guidelines and regulations and upon consultation as well as under supervision of the responsible Ethics Committee of the Medical Faculty of the University of Heidelberg. According to the Ethics Committee, studies using strictly anonymized human sample material are not subject to the duty of referral to and consultation with the Ethics Committee.

### RNA secondary structure calculation

The secondary structure of RNAs was calculated by RNAfold algorithm of the Vienna RNA package^78^ using default settings.

### Sequence alignment and tree generation

For sequence comparison, DNA sequences were clustered using ClustAl W algorithm (version 2.09)^79^ with default parameters. The alignment and the ClustAl W guide tree describing the approximate groupings of the sequences by similarity is shown.

### Data availability

RNA-Seq data have been deposited at NCBI Gene Expression Omnibus database (https://www.ncbi.nml.nih.gov/geo) under accession number GSE98192.

## Electronic supplementary material

Supplemental Material

## References

[1] WCRF World Cancer Research Fund, Food, nutrition, physical activity, and the prevention of cancer: a global perspective. *AICR*, (2007).

[2] Chan DS (2011). Red and processed meat and colorectal cancer incidence: meta-analysis of prospective studies. Plos One.

[3] Corpet DE (2011). Red meat and colon cancer: should we become vegetarians, or can we make meat safer?. Meat. Sci..

[4] Huxley RR (2009). The impact of dietary and lifestyle risk factors on risk of colorectal cancer: a quantitative overview of the epidemiological evidence. Int. J. Cancer.

[5] Egeberg R (2008). Meat consumption, N-acetyl transferase 1 and 2 polymorphism and risk of breast cancer in Danish postmenopausal women. Eur. J. Cancer Prev..

[6] Ferrucci LM (2009). Intake of meat, meat mutagens, and iron and the risk of breast cancer in the prostate, lung, colorectal, and ovarian cancer screening trial. Br. J. Cancer.

[7] Pala. V (2009). Meat, eggs, dairy products, and risk of breast cancer in the european prospective investigation into cancer and nutrition (EPIC) cohort. Am. J. Clin. Nutr..

[8] Taylor VH, Misra M, Mukherjee SD (2009). Is red meat intake a risk factor for breast cancer among premenopausal women?. Breast Cancer Res. Treat..

[9] Ji J, Sundquist J, Sundquist K (2015). Lactose intolerance and risk of lung, breast and ovarian cancers: aetiological clues from a population-based study in Sweden. Br. J. Cancer.

[10] zur Hausen H, de Villiers EM (2015). Dairy cattle serum and milk factors contributing to the risk of colon and breast cancers. Int. J. Cancer.

[11] zur Hausen, H., Bund, T. & de Villiers, E.M., Infectious agents in bovine meat and milk and their potential role in cancer and other chronic diseases. *Curr*. *Top*. *Microbiol*. *Immunol*., in press (2017). 10.1007/82_2017_3 28349283

[12] Murray JT (1976). An unusual occurrence of multiple sclerosis in a small rural community. Can. J. Neurol..

[13] Sepcic J, Mesaros E, Materljan E, Sepic-Grahovac D (1993). Nutritional factors and multiple sclerosis in Gorski Kotar, Croatia. Neuroepidem..

[14] Malosse D, Perron H (1993). Correlation analysis between bovine populations, other farm animals, house pets, and multiple sclerosis prevalence. Neuroepidem..

[15] Christiensen JC (1975). Multiple sclerosis: some epidemiological clues to etiology. Acta Neurol. Latinoam..

[16] Ragnedda G (2015). Reduced duration of breastfeeding is associated with a higher risk of multiple sclerosis in both Italian and Norwegian adult males: the EnvIMS study. J. Neurol..

[17] Poorolajal J (2015). Multiple sclerosis associated risk factors: a case-control study. Iran. J. Public Health.

[18] Sugimura, T. *et al*., Mutagen carcinogens in food, with special reference to highly mutagenic pyrolytic products in broiled foods. *Cold Spring Harbor*, 156177 (1977).

[19] Sugimura T, Wakabayashi K, Nakagama H, Nagao M (2004). Heterocyclic amines: mutagens/ carcinogens produced during cooking of meat and fish. Cancer Sci..

[20] Ohgaki H (1986). Carcinogenicity in mice and rats of heterocyclic amines in cooked foods. Environ. Health Perspect..

[21] Yano M (1988). Presence of nitrosable mutagen precursors in cooked meat and fish. Mutat. Res..

[22] Knize MG, Salmon CP, Pais P, Felton JS (1999). Food heating and the formation of heterocyclic aromatic amine and polycyclic aromatic hydrocarbon mutagens/carcinogens. Adv. Exp. Med. Biol..

[23] zur Hausen H (2012). Red meat consumption and cancer: reasons to suspect involvement of bovine infectious factors in colorectal cancer. Int. J. Cancer.

[24] zur Hausen H (2015). Risk factors: what do breast and CRC cancers and MS have in common?. Nat. Rev. Clin. Oncol..

[25] Whitley C (2014). Novel replication-competent circular DNA molecules from healthy cattle serum and milk and multiple sclerosis-affected human brain tissue. Genome Announc..

[26] Funk M (2014). Isolation of protein-associated circular DNA from healthy cattle serum. Genome Announc..

[27] Falida K, Eilebrecht S, Gunst K, zur Hausen H, de Villiers EM (2017). Isolation of two virus-like circular DNAs from commercially available milk samples. Genome Announc..

[28] Manuelidis L (2011). Nuclease resistant circular DNAs copurify with infectivity in scrapie and CJD. J. Neurovirol..

[29] Chattoraj DK (2000). Control of plasmid DNA replication by iterons: no longer paradoxical. Mol. Microbiol..

[30] Matsunaga F (1995). DNA-binding domain of the RepE initiator protein of mini-F plasmid: involvement of the carboxyl-terminal region. J. Bacteriol..

[31] Povilonis J (2013). Spread of carbapenem-resistant Acinetobacter baumannii carrying a plasmid with two genes encoding OXA-72 carbapenemase in Lithuanian hospitals. J. Antimicrob. Chemother..

[32] Lamberto I, Gunst K, Müller H, zur Hausen H, de Villiers EM (2014). Mycovirus-like DNA virus sequences from cattle serum and human brain and serum samples from multiple sclerosis patients. Genome Announc..

[33] Gunst K, zur Hausen H, de Villiers EM (2014). Isolation of bacterial plasmid-related replication-associated circular DNA from a serum sample of a multiple sclerosis patient. Genome Announc..

[34] Buck CB, Pastrana DV, Lowy DR, Schiller JT (2004). Efficient intracellular assembly of papillomaviral vectors. J. Virol..

[35] Buck CB, Pastrana DV, Lowy DR, Schiller JT (2005). Generation of HPV pseudovirions using transfection and their use in neutralization assays. *Meth*. Mol. Med..

[36] de Villiers EM, Borkosky SS, Kimmel R, Gunst K, Fei JW (2011). The diversity of torque teno viruses: *in vitro* replication leads to the formation of additional replication-competent subviral molecules. J. Virol..

[37] Dobin, A. *et al*., STAR: ultrafast universal RNA-seq aligner. *Bioinform*. **29**, 15–21 (2013). 10.1093/bioinformatics/bts635 PMC3530905 23104886

[38] Borkosky SS, Whitley C, Kopp-Schneider A, zur Hausen H, de Villiers EM (2012). Epstein-Barr virus stimulates torque teno virus replication: a possible relationship to multiple sclerosis. PLoS One.

[39] Qin LQ (2004). Milk consumption is a risk factor for prostate cancer: meta-analysis of case-control studies. Nutr. Cancer.

[40] Snowdon DA, Phillips RL, Choi W (1984). Diet, obesity, and risk of fatal prostate cancer. Am. J. Epidemiol..

[41] Qin LQ, Xu JY, Wang PY, Tong J, Hoshi K (2007). Milk consumption is a risk factor for prostate cancer in western countries: evidence from cohort studies. Asia Pac. J. Clin. Nutr..

[42] Chan JM, Gann PH, Giovannucci EL (2005). Role of diet in prostate cancer development and progression. J. Clin. Oncol..

[43] Park M (2005). Consumption of milk and calcium in midlife and the future risk of Parkinson disease. Neurology.

[44] Chen H (2007). Consumption of dairy products and risk of Parkinson’s disease. Am. J. Epidemol..

[45] Chade AR, Kasten M, Tanner CM (2006). Nongenetic causes of Parkinson’s disease. J. Neural. Transm. Suppl..

[46] Yeh, Y.H., Gunasekharan, V. & Manuelidis, L. A prokaryotic viral sequence is expressed and conserved in mammalian brain. *Proc*. *Natl*. *Acad*. *Sci*. *USA*, 10.1073/pnas (2017). 10.1073/pnas.1706110114 PMC5502646 28630311

[47] Shaw G, Morse S, Ararat M, Graham FL (2002). Preferential transformation of human neuronal cells by human adenoviruses and the origin of HEK 293 cells. FASEB J..

[48] Stutika C (2015). A comprehensive RNA sequencing analysis of the adeno-associated virus (AAV) type 2 transcriptome reveals novel AAV transcripts, splice variants, and derived proteins. J. Virol..

[49] Lamontagne J, Mell JC, Bouchard MJ (2016). Transcriptome-wide analysis of hepatitis B virus-mediated changes to normal hepatocyte gene expression. PLoS Pathog..

[50] Wegrzyn K (2014). Sequence-specific interactions of Rep proteins with ssDNA in the AT-rich region of the plasmid replication origin. Nucleic Acids Res..

[51] Grabe N (2002). AliBaba2: context specfic identfication of transcription factor binding sites. In Silico Biol..

[52] Perry RP (2005). The architecture of mammalian ribosomal protein promoters. BMC Evol. Biol..

[53] Core LJ, Waterfall JJ, Lis JT (2008). Nascent RNA sequencing reveals widespread pausing and divergent initiation at human promoters. Science.

[54] Seila AC (2008). Divergent transcription from active promoters. Science.

[55] Melgar MF, Collins FS, Sethupathy P (2011). Discovery of active enhancers through bidirectional expression of short transcripts. Genome Biol..

[56] Sigova AA (2013). Divergent transcription of long noncoding RNA/mRNA gene pairs in embryonic stem cells. Proc. Natl. Acad. Sci. USA.

[57] Wu X, Sharp PA (2013). Divergent transcription: a driving force for new gene origination?. Cell.

[58] Beaudoing E, Freier S, Wyatt JR, Claverie JM, Gautheret D (2000). Patterns of variant polyadenylation signal usage in human genes. Genome Res..

[59] Weindler FW (1991). & Heilbronn, R., A subset of herpes simplex virus replication genes provides helper functions for productive adeno-associated virus replication. J. Virol..

[60] Buller RM, Janik JE, Sebring ED, Rose JA (1981). Herpes simplex virus types 1 and 2 completely help adenovirus-associated virus replication. J. Virol..

[61] Rosario K, Duffy S, Breitbart M (2012). A field guide to eukaryotic circular single-stranded DNA viruses: insights gained from metagenomics. Arch. Virol..

[62] Cohen DR, Curran T (1989). The structure and function of the fos proto-oncogene. Crit. Rev. Oncog..

[63] Cappello F, Bellafiore M, David S, Anzalone R, Zummo G (2003). Ten kilodalton heat shock protein (HSP10) is overexpressed during carcinogenesis of large bowel and uterine exocervix. Cancer Lett..

[64] Cappello F (2007). Hsp60 and Hspl0 as antitumor molecular agents. Cancer Biol. Ther..

[65] Melle C (2006). Detection and identification of heat shock protein 10 as a biomarker in colorectal cancer by protein profiling. Proteomics.

[66] Berggren M (1996). Thioredoxin and thioredoxin reductase gene expression in human tumors and cell lines, and the effects of serum stimulation and hypoxia. Anticancer Res..

[67] Raffel J (2003). Increased expression of thioredoxin-1 in human colorectal cancer is associated with decreased patient survival. J. Lab. Clin. Med..

[68] Opavsky R (2007). Specific tumor suppressor function for E2F2 in Myc-induced T cell lymphomagenesis. Proc. Natl. Acad. Sci. USA.

[69] Pusapati RV, Weaks RL, Rounbehler RJ, McArthur MJ, Johnson DG (2010). E2F2 suppresses Myc-induced proliferation and tumorigenesis. Mol. Carcinog..

[70] Morrison JA (2014). Thioredoxin interacting protein (TXNIP) is a novel tumor suppressor in thyroid cancer. Mol. Cancer.

[71] Morgan NV (2006). PLA2G6, encoding a phospholipase A2, is mutated in neurodegenerative disorders with high brain iron. Nat. Genet..

[72] Kurian MA (2008). Phenotypic spectrum of neurodegeneration associated with mutations in the PLA2G6 gene (PLAN). Neurology.

[73] Kim D (2013). TopHat2: accurate alignment of transcriptomes in the presence of insertions, deletions and gene fusions. Genome Biol..

[74] Li H (2009). The Sequence alignment/map (SAM) format and SAMtools. Bioinformatics.

[75] Anders S, Pyl PT, Huber W (2014). HTSeq–a Python framework to work with high-throughput sequencing data. Bioinformatics.

[76] Robinson MD, McCarthy DJ, Smyth GK (2010). edgeR: a bioconductor package for differential expression analysis of digital gene expression data. Bioinformatics.

[77] McCarthy JD, Chen Y, Smyth KG (2012). Differential expression analysis of multifactor RNA-Seq experiments with respect to biological variation. Nucl. Acid. Res..

[78] Gruber AR, Lorenz R, Bernhart SH, Neuböck R, Hofacker IL (2008). The vienna RNA websuite. Nucl. Acid. Res..

[79] Thompson JD, Higgins DG, Gibson TJ, CLUSTAL W (1994). improving the sensitivity of progressive multiple sequence alignment through sequence weighting, position-specific gap penalties and weight matrix choice. Nucl. Acid. Res..

