# Supplementary material for: Expression and replication of virus-like circular DNA in human cells
Source: Sci Rep. 2018 Feb 12;8:2851. doi: 10.1038/s41598-018-21317-w (PMC5809378; doi:10.1038/s41598-018-21317-w)

Expression and replication of virus-like circular DNA in human cells

Sebastian Eilebrecht, Agnes Hotz-Wagenblatt, Victor Sarachaga, Amelie Burk, Konstantina Falida, Deblina Chakraborty, Ekaterina Nikitina, Claudia Tessmer, Corinna Whitley, Charlotte Sauerland, Karin Gunst, Imke Grewe and Timo Bund

Supplemental material

Reverse transcription (RT) PCR and long PCR.

For cDNA synthesis 1µg of DNase1-treated total RNA was incubated with either 1µl random hexamer (20µM) or oligo-dT primers (12µM) at 72°C for 3 minutes, before the reaction was cooled to 42°C for 2 minutes. After adjustment to 1x First-Strand Buffer, 2.5mM DTT and 1mM dNTPs, 20 U RNase Inhibitor (Clontech) and 100 U SMARTScribe Reverse Transcriptase (Clontech) were added and the reaction was incubated at 42°C for 90 minutes. Subsequently the reaction was terminated by incubation at 70°C for 10 minutes and the total volume was adjusted to 200µl with Tricine-EDTA buffer (Clontech). A reaction lacking reverse transcriptase served as a negative control (-RT). For the detection of cDNAs of polyadenylated transcripts of MSBI1.176, 2.5µl of an oligo-dT primed RT reaction were amplified by PCR using SeqAmp DNA Polymerase (Clontech) as recommended by the manufacturer in the presence of the primers MSBI1.176 F384: 5'-CTT TTA AAG TTT TTA AAT GCT TTT AAA TGC-3' and R1693 5'-CCC TGA TTA CAG TGT TTT TGC TTT TG-3', F647: 5’-TAT AAC TTA GCT TTG GTT GAA CAG A-3’ and R1303: 5’-CGT TTT ATC GCT GTT TTG TTT CTT G-3’ or F28: 5’-AGC GAC TCA ATG AAA GTT CGA TTA TTC CCC-3’ and R1693. 1 ng of circular MSBI1.176 DNA was used as a template for a positive control reaction for each primer combination. The PCR program was as following: 30 cycles: 94°C for 30 seconds, 60°C for 30 seconds, 72°C for 3 minutes. For the detection of cDNAs of

polyadenylated transcripts of CMI1.252, 2.5 µl of an oligo-dT primed RT reaction were amplified as mentioned above in the presence of the primers CMI1.252 F352: 5'-CTT TTA AAG TTT TTA AAT GCT TTT AAA TGC-3' and R2143: 5'-GGT TGT CCA CGA TAC TCA CTT TT-3', F1510: 5'-AAA ACA AGC TAG AAG AAT TTG GCG-3' and R2281: 5'-CTA CAT TGT TCA GGC TTT AGC TC-3' or F57: 5'-TAC ACG AGC AAA GCG AGT TCA TA-3' and R2143.

Long PCR for detection of full length MSBI1.176 or CMI1.252 DNA was performed using La Taq polymerase (Takara) as recommended by the manufacturer. 1 ng of DpnI-digested circular MSBI1.176 or CMI1.252 input DNA was used as a template for a negative control reaction and 1 ng of non-digested input DNA was used as a template for a positive control reaction. The corresponding primers were MSBI1.176 F1656: 5'-CCC CTC AAC CCC AAA AGC AA-3' and MSBI1.176 R1655: 5'-TTT ACC CCC ACG GAC TTC CA-3' and CMI1.252 F2250: 5'-CAA CAA TGA GCG AGC TAA AGC C-3' and R 2249: 5'-TAC GAA AGC TAG CAC CAC CG-3'. The long PCR program for MSBI1.176 detection was as following: 94°C for 1 minute, 5 cycles: 94°C for 30 seconds, 58°C for 1 minute, 72°C for 4 minutes, 5 cycles: 94°C for 30 seconds, 56°C for 1 minute, 72°C for 4 minutes, 20 cycles: 94°C for 30 seconds, 54°C for 1 minute, 72°C for 4 minutes seconds, 72°C for 10 minutes. For CMI1.252 detection the following PCR program was used: 94°C for 1 minute, 5 cycles: 94°C for 30 seconds, 66°C for 1 minute, 72°C for 4 minutes, 5 cycles: 94°C for 30 seconds, 64°C for 1 minute, 72°C for 4 minutes, 20 cycles: 94°C for 30 seconds, 62°C for 1 minute, 72°C for 4 minutes seconds, 72°C for 10 minutes.

Quantitative PCR (qPCR).

Quantitative PCR (qPCR) analyses were performed using the SYBR Green PCR Master Mix (Applied Biosystems) and a Stratagene Mx3000P qPCR machine (Stratagene) as recommended by the manufacturer. For the quantification of RT-generated cDNA the following primers were used: CMI1.252 F1514 5'-ACA AGC TAG AAG AAT TTG GCG TG-3', CMI1.252 R1734 5'-TGG CAC AGT AGA GCT TTC ATC A-3', CMI3.168 F647 5'-AGG CGA GAG AAA CAG GCA AA-3', CMI3.168 R851 5'-TGC GAT ACC CAA CGA CTT GT-3', MSBI1.176 F433 5'-CCC ACA CAG CAA GGC ATA CA-3', MSBI1.176 R717 5'-CCT TTG CCT GTT TCT CTC GC-3', MSBI2.176 F1216 5'-TCA CAG CCA CCT ACG AAC AG-3', MSBI2.176 R1381 5'-TTT GCG ATC TGA GGC TCT GT-3', beta actin F 5'-CCA CCA TGT ACC CTG GCA TT-3' and beta actin R 5'-ATC TGA GGA GGG AAG GGG AC-3'. For the genome-specific quantification of DpnI-resistent, replicated DNA the following primers were used: MSBI1.176 F235 5'-ACG GGT AGG CTT GCT TAT TTG A-3', MSBI1.176 R717 5'-CCT TTG CCT GTT TCT CTC GC-3', CMI1.252 F1514 5'-ACA AGC TAG AAG AAT TTG GCG TG-3' and CMI1.252 R1734 5'-TGG CAC AGT AGA GCT TTC ATC A-3'. Notably, the latter primer combinations cover at least two DpnI restriction sites in either genome and are isolate-specific. Absolute amounts of replicated CMI1.252 and MSBI1.176 DNA molecules were quantified using standard curves with defined amounts of the corresponding circular molecules.

Rapid amplification of cDNA ends (RACE).

To generate 5'- and 3'-RACE-ready cDNA, the SMARTer RACE 5'3' kit (Clontech) was used. For 5'-RACE-ready cDNA, 1µg of DNase1-treated total RNA from transfected HEK293TT cells was incubated with 1µl of primer MSBI1.176 RT848 (5'-GGT AAC TGA ATT GAC GGG CA-3'), MSBI1.176 RT1296 (5'-TCG CTG TTT TGT TTC TTG TGC-3') or

MSBI1.176 RT1431 (5'-ATT TCC CCT GTA ACA CGG CT-3') (2.5µM) at 72°C for 3 minutes, before the reaction was cooled to 42°C for 2 minutes. In the case of CMI1.252, the primers CMI1.252 RT1052 (5’-TTT TTC CTG TGC TAC GCC AA-3’) or CMI1.252 RT2095 (5’- ATG CTA CAA AAC CAA CGC CA-3’) were used. After addition of 1µl of the SMARTer II A oligonucleotide (24µM), the reaction was adjusted to 1x first-strand buffer, 2.5mM DTT and 1mM dNTPs, then 20 U RNase Inhibitor (Clontech) and 100 U SMARTScribe Reverse Transcriptase (Clontech) were added and the reaction was incubated at 42°C for 90 minutes. Subsequently the reaction was terminated by incubation at 70°C for 10 minutes and the total volume was adjusted to 200µl with Tricine-EDTA buffer (Clontech). 3'-RACE-ready cDNA was prepared from 1µg of DNase1-treated total RNA from transfected HEK293TT cells as recommended by the manufacturer. For RACE, 2.5µl of 5'- or 3'-RACE-ready cDNA was used as a template for a PCR reaction containing 1µl 5'- or 3'- gene specific primer (10µM), 1x universal primer mix (UPM) and 1µl SeqAmp DNA Polymerase in 1x SeqAmp buffer (Clontech). The PCR program was as following: 5 cycles: 94°C for 30 seconds, 72°C for 3 minutes; 5 cycles: 94°C for 30 seconds, 70°C for 30 seconds, 72°C for 3 minutes; 20 cycles: 94°C for 30 seconds, 68°C for 30 seconds, 72°C for 3 minutes. Primers used for 5'- RACE of MSBI1.176 were: MSBI1.176 R813 5'-GCA TCT TTG AGG GCT TGA TAT GCC GT-3', MSBI1.176 R1258 5'-TCC TGT AAT CAC TCG CCC TTT TTT GTG T-3' and MSBI1.176 R1383 5'-GCG TTT TCA GGC TGT TTT ACA ATG TTG-3'. Nested 5’-RACE of CMI1.252 were performed using the following primers: CMI1.252 R724 5’- GCA TGA ACT GTT AAG GGG TCA TTG GC-3’, CMI1.252 R920 5’- CAG GGG CAA AAA TAA TTT CAA CGG TTG-3’, CMI1.252 R1883 5’- GGG TTC TTC GCT TTG TCT TCT AAC ATA TTG CT-3’ and CMI1.252 R2005 5’- CAG CAG CTT CTT GAT TCT CCA ACA TAG TCA-3’. Primers used for nested 3'-RACE of MSBI1.176 were: MSBI1.1 76 F788 5'-ACG GCA TAT CAA GCC CTC AAA GAT GC-3', MSBI1.176 F944 5'-GCC CCT

GCG GTT GTT CCT CTG ATT ACA-3', MSBI1.176 F1655 5'-ACC CCT CAA CCC CAA AAG CAA AAA CA-3' and MSBI1.176 F155 5'-TGC TTT TGC TTG TTT TCG GGT CTT AGG GG-3'. Nested 3'-RACE of CMI1.252 were performed using the following primers: CMI1.252 F695 5'- GCC AAT GAC CCC TTA ACA GTT CAT GCA AGT-3', CMI1.252 F894 5'- CAA CCG TTG AAA TTA TTT TTG CCC CTG-3', CMI1.252 F1852 5'- AGC AAT ATG TTA GAA GAC AAA GCG AAG AAC CC-3' and CMI1.252 F1976 5'- TGA CTA TGT TGG AGA ATC AAG AAG CTG CTG-3'. For pRACE cloning all primers were equipped with the sequence 5'-GAT TAC GCC AAG CTT-3' at their 5'-end. The PCR program was as following: 5 cycles: 94°C for 30 seconds, 72°C for 3 minutes; 5 cycles: 94°C for 30 seconds, 70°C for 30 seconds, 72°C for 3 minutes; 25 cycles: 94°C for 30 seconds, 68°C for 30 seconds, 72°C for 3 minutes. Resulting RACE fragments were separated by agarose gel electrophoresis, eluted using the NucleoSpin gel cleanup kit (Machery & Nagel), subcloned into the pRACE vector (Clontech) as recommended by the manufacturer's and identified by conventional Sanger sequencing. For MSBI1.176 a total of 497 clones were sequenced (primer R813: 128 clones, primer R1258: 108 clones, primer R1383: 167 clones, primer F944: 94 clones). For CMI1.252 a total of 384 clones were sequenced (primer R724: 96 clones, primer R1883: 108 clones, primer F894: 120 clones, primer F1976: 60 clones). RACE reactions using cDNAs generated from DNase1-treated total RNA of mock transfected HEK293TT cells were used as negative controls. To exclude PCR artifacts, an additional control was included, in which 1 ng of linearized BMMF DNA was used as a template for RACE reactions.

Synthesis of northern blot probes

Strand-specific RNA probes for northern blot analyses were generated by *in vitro* transcription of double stranded DNA templates containing a T7 promoter using the

TranscriptAid T7 High Yield Transcription Kit (Thermo Scientific) in the presence of 1 mM Digoxigenin-11-UTP (Roche) as recommended by the manufacturer. Probe integrity was assessed by Bioanalyzer technology (Applied Biosystems).

Northern blot analyses.

For northern blot analyses 5µg of total cellular RNA were denatured for 5 minutes at 68°C in the presence of 25% Formamide, 1.8M Formaldehyde and 10 mg/ml Ficoll in MOPS buffer (20mM 3-Morpholinopropane-1-sulfonic acid, 5mM NaOAc, 1mM Ethylendiamintetra-acetic acid disodium salt, pH 7.0) and subsequently loaded on a 1% denaturing Formaldehyde agarose gel containing 1.8M Formaldehyde in MOPS buffer. Electrophoresis was performed for 2.5 hours at 80V using MOPS buffer containing 80mM Formaldehyde as running buffer. RiboRuler High Range RNA Ladder (Fermentas) was used as a size marker, which was visualized by Ethidium Bromide staining prior to blotting. After electrophoresis the gel was washed in 10xSaline Sodium Citrate (SSC) buffer (Fisher Bioreagents) and diffusion-transferred to a Hybond N+ Nylon membrane (Amersham) for 16 hours. After transfer the membrane was washed twice with 2xSSC buffer, before the nucleic acids were crosslinked to the membrane by UV exposure (Stratalinker 1800, 120.000µJ). The membrane was pre-hybridized in ULTRAhyb® buffer (Ambion) at 42°C for 6 hours and subsequently hybridized with 100ng/ml Digoxigenin-labeled RNA probe in ULTRAhyb® buffer at 42°C for 16 hours. After hybridization the membrane was washed twice for 5 minutes with 2xSSC containing 0.1% SDS and once for 15 minutes with 0.1xSSC containing 0.1% SDS at 42°C. The membrane was incubated in washing buffer (0.1M Maleic acid, 150mM NaCl, 0.3% Tween 20, pH 7.5) for 2 minutes and subsequently blocked with blocking buffer (1% skim milk in 0.1M Maleic acid, 150mM NaCl, pH 7.5) for 45 minutes before incubating with anti-Digoxigenin-AP Fab fragments (Roche) at a dilution of 1:10.000 in blocking buffer for 1

hour. Then the membrane was washed twice for 15 minutes with washing buffer and equilibrated for 3 minutes with equilibration buffer (0.1M Tris-HCl, 100 mMNaCl, pH 9.5). For development the membrane was incubated with CPD-Star reagent (Applied Biosystems) for 5 minutes, before signals were detected on a BioRad WesternBlot detection system.

Protein purification for ELISAs.

Chemically competent *E. coli* (SoluBl21, Genlantis) were transformed with a MSBI1.176 Rep expression plasmid (pEXP5-CT, Invitrogen) followed by clonal selection of high level protein expression colonies on LB-agar plates containing Ampicillin. The colony was then used to inoculate 1000 ml LB medium containing Ampicillin and grown until an OD600 of 0.5 was reached. Protein expression was induced by IPTG (0.66mM) over night at 25°C on a shaking device, before cells were centrifuged at 6.000xg and 4°C, washed with PBS and re-dissolved by sonication in 20 packed cell volumes of sample buffer (100mM NaH_2PO_4, 10mM Tris-HCl, 5 mM Imidazole, 5mM beta-Mercaptoethanol, pH 8.0) containing 8M Urea. The protein was subsequently purified at denaturing conditions (55mM Imidazole for washing and 300mM Imidazole for protein elution) via a C-terminal His_6-tag by affinity chromatography (Clontech His60 Ni Superflow resin). The protein purification was followed by Coomassie protein staining and Rep protein purity was calculated densitometrically.

Production of mouse monoclonal anti-Rep antibodies.

Mouse monoclonal antibodies were produced by individual immunizations of mice (DKFZ, in house) with two different KLH-coupled synthetic peptides (peptide 1: CEARETGKGINANDPLTVH; peptide 2: CKQINEHTDITASYEQHKKGRT, PSL GmbH, Heidelberg) or with purified, full length MSBI1 Rep protein obtained by denaturing His-tag affinity purification out of E. coli. Rep-specificity of the double sub-cloned hybridomas was

verified by western blotting, immunofluorescence microscopy, immunoprecipitation and ELISA experiments based on detection of Rep fusion proteins with NanoLuc (Promega), ZsGreen1CI (Clontech) or 3xFlag (Sigma Aldrich) fusion tags as expression controls.

Neutralization ELISAs.

ELISA neutralization assays were performed as triplicate experiment to verify the binding specificity of human plasma antibodies for the Rep antigens analysed in standard ELISA serological experiments (duplicates within each experiment). The experimental setup included all the experimental steps previously described for the standard ELISA assay, but, in addition, included an incubation step (1 h at 37 °C) with a dilution series of a pool of 10 individual, affinity purified Rep-reactive mouse monoclonal antibodies (1:25-1:1600 in superblock assay buffer) after antigen coating and blocking. After binding of mouse anti-Rep antibodies and washing, plasma incubations as well as serological read out with anti-Human IgG HRP-coupled secondary antibodies was performed as described previously. Specific binding of anti-Rep mouse antibodies was monitored by parallel experiments confirming Rep antigen binding with a mouse-specific detection antibody (also from Dianova, MinX).

Supplemental figure legends

Figure S1.

(A) RT-qPCR assessment of +RT/-RT controls of CMI1.252, CMI3.168, MSBI1.176 and MSBI2.176 in HEK293TT cells. Circular CMI1.252, CMI3.168, MSBI1.176 and MSBI2.176 was transfected into HEK293TT cells, total RNA was isolated 72 hours post transfection and subjected to reverse transcription using random hexamer primers (+RT) followed by qPCR quantification of the resulting cDNA using isolate-specific (left) and beta actin (right) primers. Reverse transcription reactions lacking the reverse transcriptase enzyme were used as

a negative control (-RT) in order to access the ratio of RNA-derived cDNA to residual input DNA in biological triplicates. Absolute quantifications were performed using a standard curve for each isolate. Signals of the +RT reactions were normalized to 100% for each isolate.

(B) RT-qPCR results from BMMFtransfected HEK293TT cells, non-transfected cells as well as water controls using each BMMF-specific primer set as indicated. qPCR results were normalized to beta actin as a housekeeping gene, amounts are displayed as -fold beta actin.

(C) Putative splicing events for the isolates CMI1.252, CMI3.168 and MSBI1.176 as detected by RNA-Seq. RNA-Seq reads of HEK293TT cells transfected with each isolate were analyzed for splicing events using the STAR mapping algorithm [35]. For each splicing event detected in at least two biological replicates, the intron start site, the intron end, the strand on which the splicing occurs as well as the maximum observed number of uniquely mapping reads are indicated.

Figure S2.

(A) MSBI1.176 replication positive control: HEK293TT cells were transfected with an MSBI1.176 genome containing the SV40 origin of replication. Absolute amounts of DpnI-sensitive (black) and DpnI-resistant (replicated, grey) DNA at days 6, 10 and 13 post transfection were quantified by qPCR.

(B) Assessment of DpnI digestion efficacy: 100 ng of MSBI1.176 or CMI1.252 were digested with DpnI enzyme as indicated in the materials and methods section of our manuscript either solely in water or in the presence of 10µg of total genomic DNA from HEK293TT cells. Reactions lacking DpnI enzyme served as controls. Relative amounts of MSBI1.176 or CMI1.252 were quantified by qPCR using DpnI-sensitive primers.

Figure S3.

(A) ClustAl W dendogram showing the similarity of the BMMFs MSBI1.176, MSBI2.176, CMI1.252, CMI3.168 and their closest related *Acinetobacter baumannii* plasmid pAB120 [80]. The relative distances are indicated.

(B) Clustering of the A/T-rich region as well as the iteron-like repeats of the BMMFs MSBI1.176, MSBI2.176, CMI1.252, CMI3.168 and the *Acineobacter baumannii* plasmid pAB120. The location of the iteron-like repeats is indicated. Nucleotide numbers correspond to the corresponding full length genomes. The color code used is based on the nucleotides, the conservation as well as the consensus sequence is given below the alignment.

Figure S4.

Comparison of putative promoter elements located within the 250 bp upstream of the major transcription start site of the Rep ORF in MSBI1.176 (upper sequence) and CMI1.252 (lower sequence). The transcription start site is indicated as an arrow. The corresponding TATA box sequence is highlighted in grey and corresponds to a tolerated variant of the TATA box identified for a large set of ribosomal protein genes [48]. Transcription factor binding sites have been identified using the Alibaba2 algorithm [47]. Transcription factor binding sites, which are in common for MSBI1.176 and CMI1.252 are highlighted in red.

Table S1.

List of genes significantly ($p<0.01$) differentially expressed upon MSBI1.176 expression in HEK293TT cells. The log(2)-fold change and the p-values are indicated.

Table S2.

List of genes significantly (p<0.01) differentially expressed upon CMI1.252 expression in HEK293TT cells. The log(2)-fold change and the p-values are indicated.

Figure S1

A

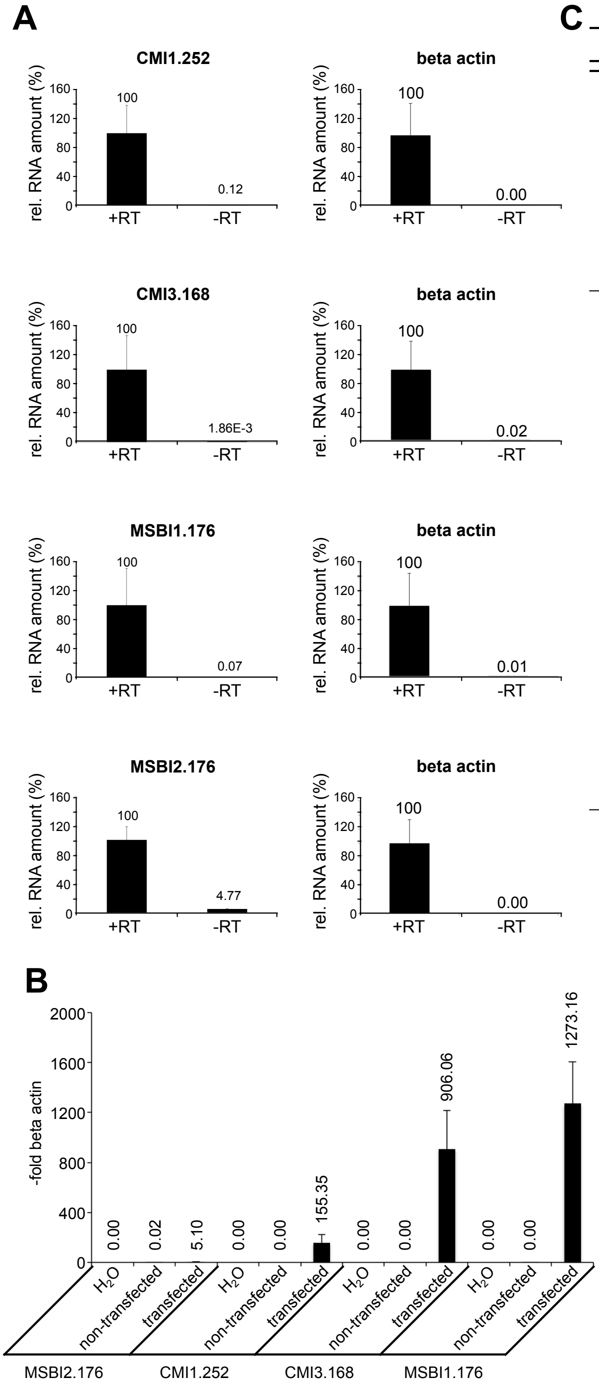

C

isolate	intron start	intron end	strand	max. unique mapping reads
**CMI1.252**	73	751	sense	2
		819	sense	3
		828	sense	1
		654	sense	1
	567	2257	sense	1
	585	2257	sense	1
	655	2251	antisense	2
	1342	2063	antisense	1
	1779	2251	antisense	1
	2098	654	sense	5
	2245	654	sense	2
	2410	2448	non-canonical	11
**CMI3.168**	132	1172	sense	19
		1227	sense	6
	343	1227	sense	4
	420	1172	sense	12
	568	1227	sense	1
	767	1172	sense	1
		1172	sense	51
	844	1227	sense	36
		1172	sense	12
	886	1172	sense	14
	907	1172	sense	1
	968	1172	sense	3
	977	1227	sense	4
	988	1172	sense	1
	1002	1172	sense	1
	1014	1395	antisense	1
	1042	1166	antisense	1
	1056	1172	sense	2
	1067	161	sense	1
		1172	sense	1
		1168	sense	2
	1073	1227	sense	11
	1075	1227	sense	5
		1172	sense	3
	1120	1227	sense	8
	1131	1227	sense	12
	1276	159	sense	3
	1403	161	sense	5
	1486	861	antisense	3
	1622	161	sense	
**MSBI1.176**	163	421	antisense	8
	280	421	antisense	7
	546	1424	sense	4
	595	1424	sense	1
	642	1422	antisense	3
		1149	antisense	4
		927	antisense	2
		708	antisense	1
		1463	antisense	1
	816	1424	sense	4
	834	1424	sense	2
	929	994	sense	3
		1537	sense	3
	985	1424	sense	2
	1068	1424	sense	3
	1105	1424	sense	4
	1122	1314	sense	2
	1344	1424	sense	11
	1364	1424	sense	1
	1550	421	antisense	2
	1659	421	antisense	1

Figure S2

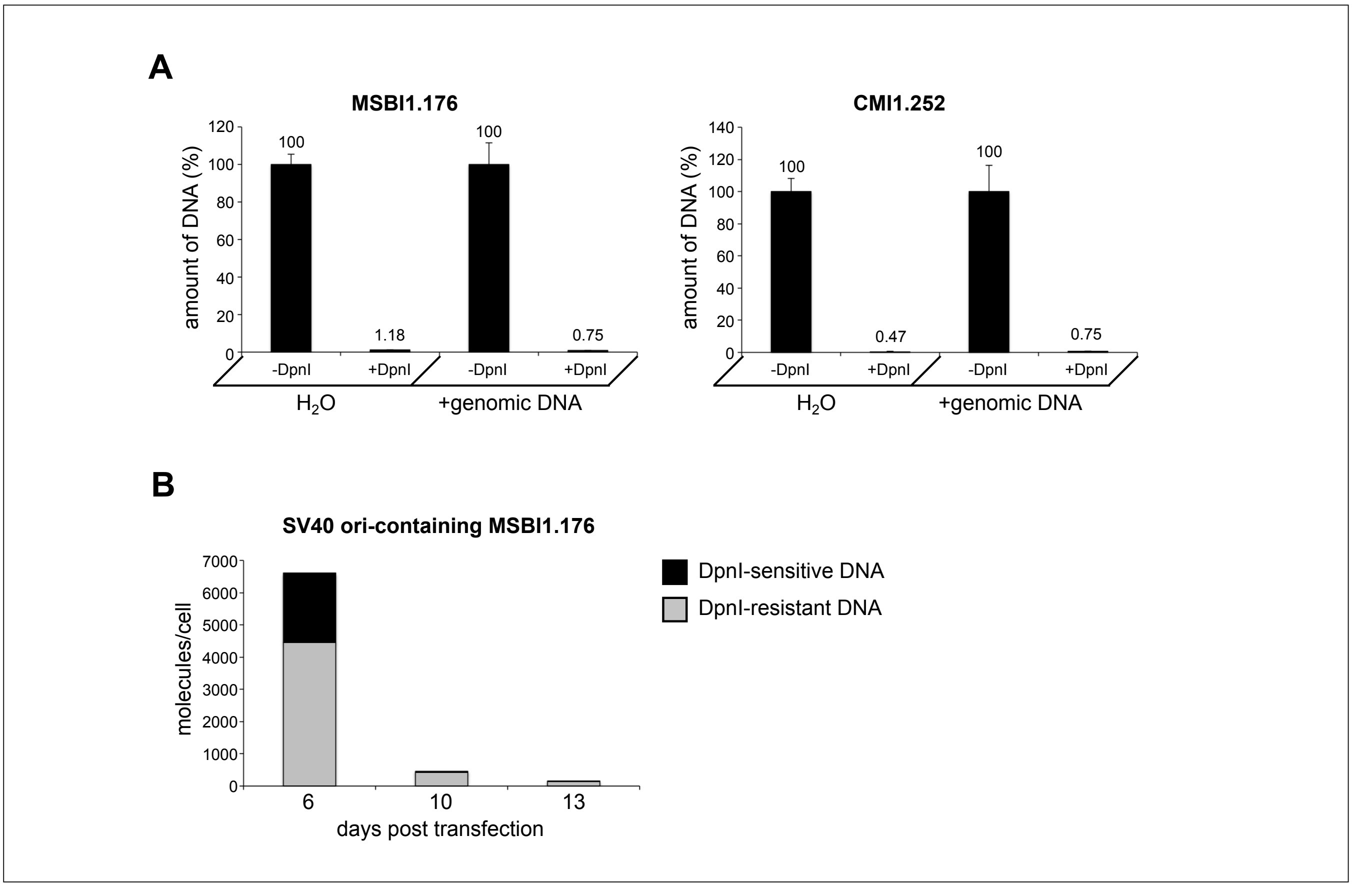
A
MSBI1.176
amount of DNA (%)
120
100
80
60
40
20
0
100
1.18
100
0.75
-DpnI
+DpnI
-DpnI
+DpnI
H2O
+genomic DNA
CMI1.252
amount of DNA (%)
140
120
100
80
60
40
20
0
100
0.47
100
0.75
-DpnI
+DpnI
-DpnI
+DpnI
H2O
+genomic DNA
B
SV40 ori-containing MSBI1.176
molecules/cell
7000
6000
5000
4000
3000
2000
1000
0
6
10
13
days post transfection
DpnI-sensitive DNA
DpnI-resistant DNA

Figure S3

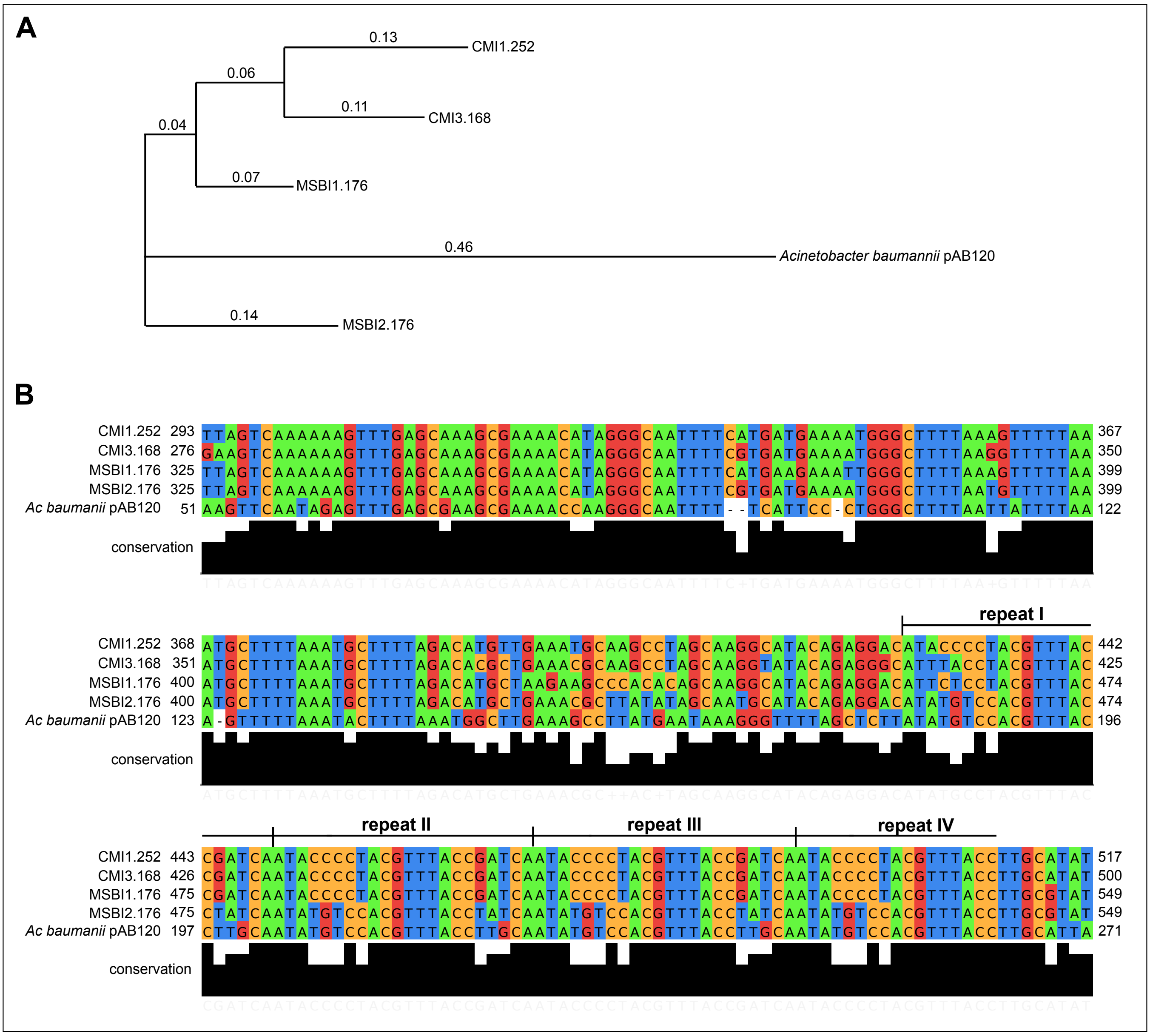

A
0.13
CMI1.252
0.06
0.11
CMI3.168
0.04
0.07
MSBI1.176
0.46
Acinetobacter baumannii pAB120
0.14
MSBI2.176
B
CMI1.252 293 … 367
CMI3.168 276 … 350
MSBI1.176 325 … 399
MSBI2.176 325 … 399
Ac baumanii pAB120 51 … 122
conservation
repeat I
CMI1.252 368 … 442
CMI3.168 351 … 425
MSBI1.176 400 … 474
MSBI2.176 400 … 474
Ac baumanii pAB120 123 … 196
conservation
repeat II
repeat III
repeat IV
CMI1.252 443 … 517
CMI3.168 426 … 500
MSBI1.176 475 … 549
MSBI2.176 475 … 549
Ac baumanii pAB120 197 … 271
conservation

Figure S4

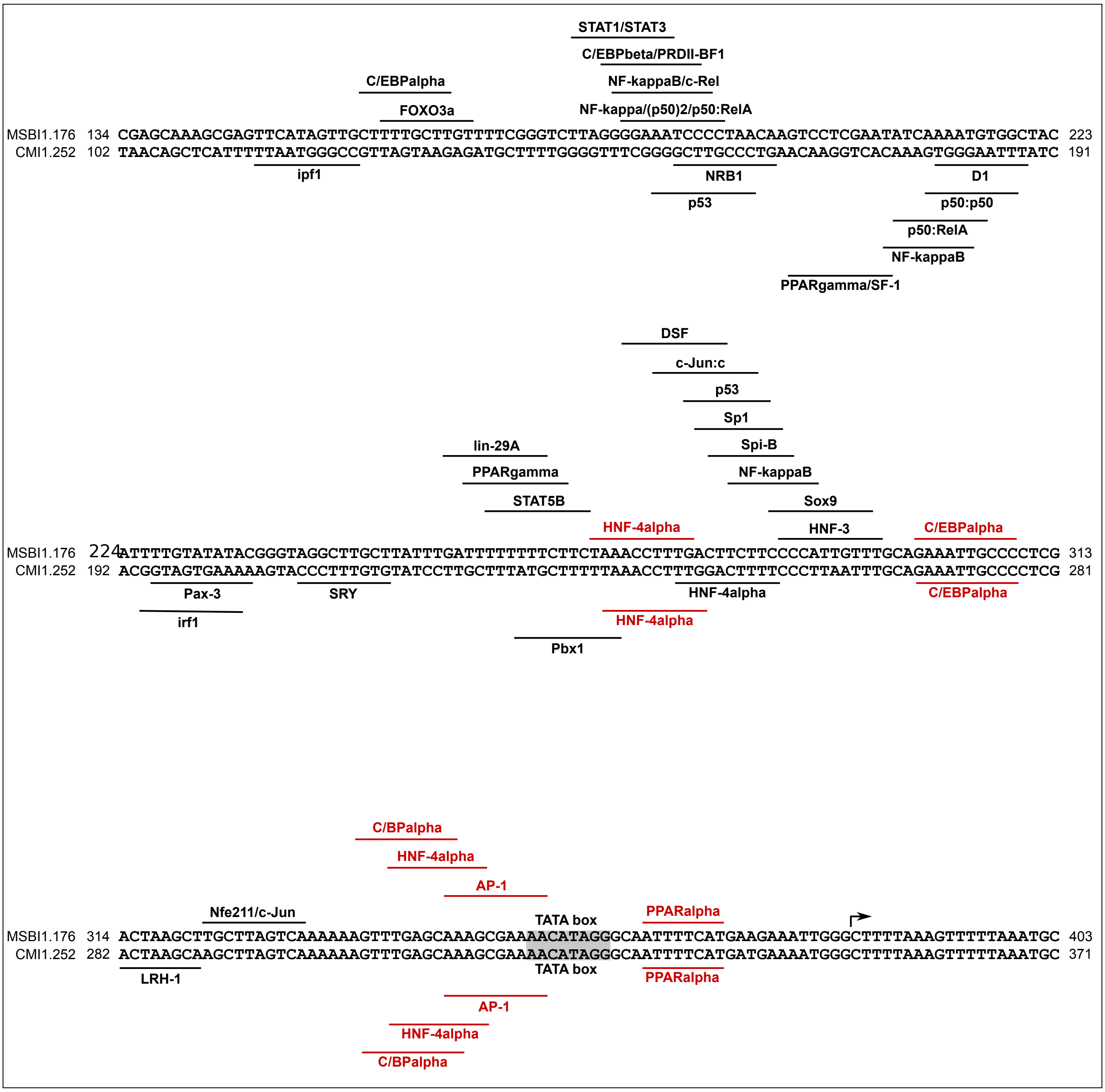
STAT1/STAT3
C/EBPbeta/PRDII-BF1
C/EBPalpha
NF-kappaB/c-Rel
FOXO3a
NF-kappa/(p50)2/p50:RelA
MSBI1.176 134 CGAGCAAAGCGAGTTCATAGTTGCTTTTGCTTGTTTTCGGGTCTTAGGGGAAATCCCCTAACAAGTCCTCGAATATCAAAATGTGGCTAC 223
CMI1.252 102 TAACAGCTCATTTTTAATGGGCCGTTAGTAAGAGATGCTTTTGGGGTTTCGGGGCTTGCCCTGAACAAGGTCACAAAGTGGGAATTTATC 191
ipf1
NRB1
D1
p53
p50:p50
p50:RelA
NF-kappaB
PPARgamma/SF-1
DSF
c-Jun:c
p53
Sp1
lin-29A
Spi-B
PPARgamma
NF-kappaB
STAT5B
Sox9
HNF-4alpha
HNF-3
C/EBPalpha
MSBI1.176 224 ATTTTGTATATACGGGTAGGCTTGCTTATTTGATTTTTTTTCTTCTAAACCTTTGACTTCTTCCCCATTGTTTGCAGAAATTGCCCCTCG 313
CMI1.252 192 ACGGTAGTGAAAAAGTACCCTTTGTGTATCCTTGCTTTATGCTTTTTAAACCTTTGGACTTTTCCCTTAATTTGCAGAAATTGCCCCTCG 281
Pax-3
SRY
HNF-4alpha
C/EBPalpha
irf1
HNF-4alpha
Pbx1
C/BPalpha
HNF-4alpha
AP-1
Nfe211/c-Jun
TATA box
PPARalpha
MSBI1.176 314 ACTAAGCTTGCTTAGTCAAAAAAGTTTGAGCAAAGCGAAAACATAGGGCAATTTTCATGAAGAAATTGGGCTTTTAAAGTTTTTAAATGC 403
CMI1.252 282 ACTAAGCAAGCTTAGTCAAAAAAGTTTGAGCAAAGCGAAAACATAGGGCAATTTTCATGATGAAAATGGGCTTTTAAAGTTTTTAAATGC 371
LRH-1
TATA box
PPARalpha
AP-1
HNF-4alpha
C/BPalpha

table S1

gene	log(2)-fold change	P-Value
FABP7	6.46	0.000402
NEUROD6	5.95	0.001435
TM4SF1	5.60	0.00309
FEZF2	5.42	0.004375
FABP4	5.24	0.002747
LINC01094	5.10	0.001001
LINC01204	4.95	0.004752
TNFSF11	4.92	0.00354
DEFB109P1	4.89	0.004337
MIR4672	4.64	0.007961
COL1A2	4.20	0.001681
SLC25A52	3.46	0.006348
VHLL	3.32	0.005596
HCAR3	2.78	0.007769
SNORD126	1.89	0.006346
EGR1	1.56	9.61E-05
SNORA71D	1.50	0.006002
SNORA74A	1.50	0.001055
FOS	1.45	0.001061
FOSB	1.39	0.004301
ADAMTSL4-AS1	1.34	0.001448
ADAM32	1.21	0.008342
C10orf32	1.15	0.007084
CYR61	1.13	0.000436
C19orf10	1.13	0.00886
POLE4	1.13	0.00306
PRC1-AS1	1.12	0.003718
U2AF1	1.11	0.000165
ATAD3B	1.10	0.008987
PSG4	1.10	0.001196
NOMO3	1.10	0.001081
JOSD2	1.09	0.008611
MRPL12	1.09	0.003548
RNU4ATAC	1.08	0.006153
EMC7	1.04	0.000751
UBE2B	1.02	0.002207
SNORA71B	1.00	0.006363
PCBD2	1.00	0.007732
PIGW	0.99	0.003734
POP1	0.94	0.004418
PSMC5	0.94	0.00093
HMBS	0.93	0.001528
TXN	0.92	0.006325
ZNF117	0.92	0.005095
RRAGC	0.89	0.008277
STAG1	0.88	0.002319
MRPL1	0.87	0.005405
POMP	0.86	0.007887

SGCB	0.85	0.006209
GTF3C6	0.85	0.003661
HSPE1	0.85	0.004931
EMILIN2	0.83	0.005564
TDP2	0.82	0.002753
BOLA3	0.82	0.005806
TMED9	0.82	0.004308
SIRT1	0.81	0.003086
OTUD1	0.81	0.006245
RRM2	0.79	0.003578
BRCC3	0.78	0.004558
TRMT10A	0.76	0.008961
KLF6	0.76	0.006387
NDUFS3	0.76	0.009084
DSTN	0.74	0.004321
ZNF480	0.74	0.009531
CTR9	0.74	0.003805
RBM3	0.74	0.00483
PSMA1	0.74	0.009903
SEPHS2	0.73	0.003852
ADI1	0.71	0.00605
HNRNPM	0.70	0.009805
MRPL15	0.65	0.008676
E2F2	-0.71	0.006255
TSPYL2	-0.72	0.007667
NIPSNAP1	-0.76	0.009554
TXNIP	-0.79	0.003394
HMGA1	-0.80	0.007529
PCED1A	-0.80	0.005222
ARHGEF6	-0.85	0.007401
AES	-0.86	0.005081
SEC31B	-0.90	0.006365
PLA2G6	-0.91	0.009329
PDK1	-0.91	0.008367
SERINC2	-0.92	0.007766
FSCN1	-0.93	0.005617
KLF7	-0.93	0.009869
CDR1	-0.95	0.009388
PLEKHB1	-0.95	0.009105
METTL7A	-0.96	0.009095
MGC12916	-0.96	0.009909
CACNB1	-0.96	0.00607
PLEKHH2	-0.98	0.001501
CYP26B1	-0.98	0.006242
NOXA1	-0.98	0.004756
CRABP2	-0.99	0.005456
IFI6	-1.00	0.003896
GABBR1	-1.01	0.005817
TRIM46	-1.01	0.003907
PRSS30P	-1.02	0.004606

SDC3	-1.02	0.005935
HSPG2	-1.02	0.006004
ZFHX2	-1.03	0.008763
LOC100652736	-1.04	0.007096
FAM131B	-1.05	0.007442
PIM1	-1.05	0.005478
BMF	-1.06	0.003037
TMCO6	-1.06	0.006823
IFITM2	-1.07	0.005081
SYT6	-1.07	0.006889
CAMK1D	-1.07	0.00859
NAV2	-1.07	0.002881
SUSD4	-1.08	0.002035
TMEM255A	-1.08	0.007569
DOC2A	-1.09	0.002639
TP53I11	-1.10	0.003542
C1orf233	-1.12	0.009433
TSPAN18	-1.12	0.005081
SEMA4G	-1.13	0.001413
GAP43	-1.15	0.004709
ATHL1	-1.15	0.000568
LIPE-AS1	-1.15	0.007323
SYT7	-1.17	0.007807
ST3GAL1	-1.17	0.00204
CEACAM1	-1.17	0.0095
PPP1R13L	-1.18	0.001203
ASS1	-1.18	0.002322
PPDPF	-1.19	0.003725
BCL11B	-1.19	0.00411
COL24A1	-1.20	0.003901
CRIP2	-1.20	0.004433
ESRRG	-1.20	0.005133
FOXD2-AS1	-1.20	0.003566
FXYD6	-1.21	0.001208
GSTM4	-1.21	0.008592
SLC16A2	-1.21	0.00096
AHNAK2	-1.22	0.000508
LPCAT4	-1.22	0.00322
NRM	-1.23	0.003113
AMT	-1.23	0.002786
PPM1J	-1.24	0.004247
SHANK2	-1.24	0.005587
LINC00086	-1.24	0.001471
SIX5	-1.24	0.001995
IFITM1	-1.25	0.001415
UNC5A	-1.25	0.009903
CTC-338M12.4	-1.25	0.002209
SLC25A35	-1.26	0.004168
NRBP2	-1.26	0.000325
PKNOX2	-1.26	0.003171

BASP1	-1.27	0.001713
HSD17B14	-1.27	0.007576
SLC30A3	-1.27	0.005977
MN1	-1.27	0.001911
TUBB4A	-1.28	0.003399
LOC100506142	-1.29	0.001796
DLX4	-1.30	0.002639
GYPC	-1.30	0.001831
PLLP	-1.31	0.003795
LOC646903	-1.31	0.006679
KCNN3	-1.32	0.002974
BAI1	-1.32	0.00135
SEMA4A	-1.32	0.009668
LINC00910	-1.33	0.004931
SPDYE3	-1.33	0.007722
USH1C	-1.33	0.005961
EFNB3	-1.35	0.000581
SLIT3	-1.36	0.005448
HLA-DOB	-1.36	0.001805
C1orf213	-1.37	0.001005
HOXC-AS1	-1.37	0.003294
FAM84B	-1.39	0.00103
CRIP3	-1.40	0.008311
GMDS-AS1	-1.41	0.003528
DENND6B	-1.42	0.000483
NLGN2	-1.43	0.000121
PADI2	-1.44	0.00279
PLEKHA6	-1.47	0.003535
TMEM171	-1.50	0.007248
NXPH4	-1.50	0.000809
PPFIA4	-1.50	0.000535
MIR3609	-1.51	0.00689
MAPK15	-1.52	0.000146
GLI1	-1.54	0.001049
PHKA2-AS1	-1.54	0.004936
LOC100272217	-1.55	0.006098
HLA-DMA	-1.56	0.002177
MRPL23-AS1	-1.56	0.004726
ADRB2	-1.57	0.005967
SLC7A3	-1.57	0.000437
SPDYC	-1.57	0.000936
IGSF21	-1.57	0.004581
DDIT4	-1.58	0.000306
CTAGE7P	-1.58	0.000202
CRHR1	-1.68	0.001519
HES7	-1.69	0.003344
SLC22A31	-1.70	0.000621
SSPO	-1.73	0.001991
MFI2-AS1	-1.77	0.001566
EDA2R	-1.80	0.005739

LOC101928689	-1.80	2.71E-05
IGFBP6	-1.84	0.008811
C19orf66	-1.87	0.009129
PCOLCE-AS1	-1.89	0.003421
ARHGAP23	-1.92	0.001525
MLC1	-1.92	0.002299
ENDOU	-1.94	0.004885
SOSTDC1	-1.95	0.008596
ZBTB32	-1.95	0.000908
PRRT2	-1.96	1.35E-05
MPZ	-1.98	0.001163
C16orf11	-1.98	0.009079
PTGES	-1.98	0.000493
C1orf95	-1.98	8.22E-05
LOC113230	-1.99	0.001844
KCNQ4	-2.00	0.003894
PRKCG	-2.01	0.000885
PACSIN1	-2.03	0.009528
LIF	-2.07	0.006503
LINC00896	-2.16	0.003691
H19	-2.20	0.007163
KRT75	-2.23	0.001872
IGFBP5	-2.25	6.13E-05
P2RY4	-2.27	0.006515
CACNG6	-2.27	0.001229
LINC00173	-2.35	0.000667
ASB11	-2.36	0.007159
DKFZP434H168	-2.43	0.006553
CACNA1I	-2.59	0.005098
IL12A-AS1	-2.61	0.008842
NUPR1	-2.62	0.00081
PNCK	-2.64	0.00125
GPR112	-2.66	0.007563
SYNDIG1L	-2.68	0.009232
LGI3	-2.75	0.004521
RHOXF1	-2.97	0.006693
CIART	-2.99	0.001059
RNF113B	-3.12	0.001587
SDR16C5	-3.21	0.009068
LRRC37A11P	-3.28	0.007061
TRH	-3.35	0.002194
PCAT6	-3.35	0.00064
FAS-AS1	-3.40	0.000191
KLK10	-3.63	0.000895
AC011738.4	-3.71	0.008404
MIR301B	-4.04	0.004559
LOC101928158	-4.08	0.003146
UPK2	-4.25	0.004323
MSLN	-4.36	0.003994
MIR196A2	-4.55	0.005243

EDN2	-4.69	0.009738
A4GNT	-4.79	0.007393
LINC00633	-4.90	0.004418
MIR4634	-4.93	0.004036
RP5-1029K10.2	-4.93	0.004671
REN	-4.99	0.005668
CRYAA	-5.08	0.008273
MIR4768	-5.11	0.005682
MIR6774	-5.16	0.003855
GADL1	-5.17	0.005322
CTAGE9	-5.23	0.003032
LOC101928767	-5.24	0.005086
MIR6870	-5.38	0.005011
LINC00943	-5.39	0.003841
LOC643355	-5.55	0.000759
DDX11L10	-5.91	0.000853

table S2

gene	log(2)-fold change	P-Value
LINC00575	4.83	0.0065572
SPANXA2-OT1	4.73	0.0072595
NCR3	4.65	0.0083563
KLRC4	4.15	0.0037143
MIR578	3.96	0.0074699
LPP-AS1	2.76	0.0039224
SNORD97	1.66	0.0044813
FOS	1.58	0.0011066
SNORA74A	1.41	0.000637
EGR1	1.37	0.0015771
SNORD45B	1.21	0.0033671
U2AF1	1.19	6.74E-05
EIF1AX-AS1	1.17	0.008158
C10orf32	1.16	0.0066349
HSPE1	1.15	0.0001766
MRPL12	1.13	0.0051451
PFDN4	1.02	0.0049079
PSG4	1.02	0.002726
HIST1H1D	1.02	0.0052125
TXN	1.02	0.0027833
HIST1H2AB	1.00	0.004968
SNRPE	0.98	0.0060926
POP1	0.95	0.0044858
TMEM126A	0.95	0.0085596
MRPL1	0.94	0.0048162
EMC7	0.92	0.0069788
UBE2B	0.91	0.0095151
GTF3C6	0.84	0.0040184
ZCCHC12	0.83	0.0085864
BOLA3	0.80	0.0073795
VBP1	0.79	0.0087598
HPRT1	0.79	0.0048921
DSTN	0.78	0.0028448
PSMC6	0.78	0.0068835
PSMC5	0.77	0.0080373
PSMA1	0.73	0.0083819
RBM3	0.73	0.0033141
RRM2	0.72	0.0082446
CCDC47	0.72	0.0064913
SIRT1	0.70	0.0080023
CENPH	0.70	0.007959
COPS2	0.68	0.0048559
MMADHC	0.66	0.0096815
IQGAP3	-0.65	0.0055072
PVRL2	-0.71	0.0079343
PAN2	-0.72	0.0051851
SREBF2	-0.80	0.0043767
LENG8	-0.81	0.0035866

PCED1A	-0.81	0.0039934
TXNIP	-0.81	0.0039243
ERF	-0.83	0.0081612
E2F2	-0.83	0.0024121
AES	-0.83	0.0050496
TBKBP1	-0.83	0.0088437
RNF44	-0.88	0.0081893
FTL	-0.91	0.0094281
RHBDD2	-0.93	0.0016784
PRR12	-0.94	0.0092897
LMOD1	-0.94	0.0059122
TMEM79	-0.94	0.0069906
HCN3	-0.97	0.004871
TMEM198B	-0.98	0.0073583
PLA2G6	-0.98	0.0040264
SEMA4G	-0.99	0.0043221
SLC22A17	-0.99	0.0075784
TPT1-AS1	-0.99	0.0080775
GABBR1	-1.00	0.0039269
EFNB3	-1.00	0.0061089
GRIP2	-1.00	0.0061548
CACNB1	-1.01	0.0041981
SEC31B	-1.02	0.0050225
SERINC2	-1.02	0.0035103
SLC25A35	-1.03	0.0075506
SLC16A2	-1.03	0.0041286
SUSD4	-1.03	0.005332
TMEM8B	-1.03	0.0074665
INO80E	-1.04	0.0067985
GRINA	-1.05	0.0035849
DENND6B	-1.06	0.0068051
CDR1	-1.07	0.0054565
MYADM	-1.08	0.0042851
DGCR11	-1.08	0.0061595
DLX4	-1.08	0.0087857
PPDPF	-1.08	0.0092484
CTC-338M12.4	-1.09	0.0095676
WNK4	-1.12	0.0037724
LOC100506142	-1.12	0.0036296
IGFBP5	-1.13	0.004886
SLC7A3	-1.13	0.0055249
COL1A1	-1.15	0.0057876
WASH5P	-1.16	0.0091126
NRBP2	-1.16	0.0023559
NLGN2	-1.16	0.0014477
GLUD1P3	-1.18	0.0072511
LOC100652736	-1.20	0.0019061
TRIM46	-1.21	0.000733
SLC6A9	-1.21	0.007884
CTAGE7P	-1.24	0.0095779

PCBP4	-1.25	0.0002551
BCAN	-1.26	0.0032855
SPDYC	-1.29	0.0035237
TRIM58	-1.30	0.0085151
SLC30A3	-1.30	0.0033087
LINC00877	-1.31	0.0072858
NR1D1	-1.31	0.0014958
KRTAP19-1	-1.33	0.0052631
PLEKHA6	-1.33	0.0076762
ASIC3	-1.34	0.0017028
WNT8B	-1.34	0.0065969
L1CAM	-1.35	0.0043219
SLC22A31	-1.36	0.0051794
CYP2E1	-1.37	0.0004016
MAPK15	-1.41	0.0005429
RFPL4A	-1.45	0.0040522
NTRK3	-1.46	0.0084716
RAPGEF3	-1.48	0.0089316
SNORD88B	-1.52	0.0009048
TMEM151A	-1.54	0.0097119
GLI1	-1.55	0.0004804
CCDC147-AS1	-1.55	0.006987
IL17RE	-1.58	0.0077628
ZBTB32	-1.59	0.0050863
KCNK9	-1.60	0.0082419
SSPO	-1.62	0.0035774
ENDOU	-1.72	0.0056333
TCERG1L	-1.73	0.0048335
ITGAL	-1.73	0.0066669
ZMAT1	-1.74	0.0089053
PRPH	-1.75	0.0084262
ATP6V1G2	-1.83	0.0008974
PRRT2	-1.85	0.000829
LIF	-1.88	0.0040283
BCYRN1	-1.89	0.0031251
SPG20OS	-1.97	0.0015627
GREM2	-1.99	0.0080243
MIR3609	-2.00	2.21E-05
ALOXE3	-2.02	0.0005102
SHISA7	-2.04	0.0016433
CIART	-2.07	0.0048897
NELL1	-2.09	0.0027595
PKD1L2	-2.16	0.0059482
VWA5A	-2.17	0.0025375
AR	-2.19	0.0004853
MPZ	-2.25	0.0012836
TCTE3	-2.35	0.0050986
SLC30A2	-2.42	0.0097051
MATN4	-2.55	0.0050881
CACNA1I	-2.64	0.0062659

RP11-67M1.1	-2.84	0.0045802
MIR3677	-3.10	0.0083205
EDA2R	-3.11	1.96E-05
CNTD2	-3.13	0.0001547
ZSCAN10	-3.42	0.0025556
DCST2	-3.49	5.41E-05
FABP5P3	-3.66	0.0038056
SPRR2F	-3.76	0.0056194
MIR1914	-3.81	0.0069007
LOC101928158	-3.86	0.0052413
GDF15	-3.87	6.45E-06
DCX	-3.87	0.0048471
ARMC12	-4.76	0.0003432
OTOP2	-4.86	0.0062546
MIR7109	-4.98	0.0093277
AC019118.2	-4.99	0.0086605
OPN5	-5.11	0.0050492
TEX36	-5.15	0.0036454
LINC01214	-5.19	0.0085767
LINC00943	-5.21	0.0058894
RBPJL	-5.23	0.0020774
BANK1	-5.43	0.0032171
LOC285768	-5.46	0.0031214
SLC6A3	-5.55	0.00338
SDPR	-5.62	0.0026785
TRIM55	-5.69	0.0016336
TAS2R40	-5.70	0.0016855

Figure 2c, loading control, full blot

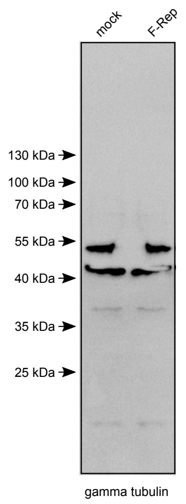

Supplement: Supplementary file 1 — Supplemental Material [file 41598_2018_21317_MOESM1_ESM.pdf]
